# *Cuphea hookeriana*: Phytochemical Profile and the Cosmeceutical and Dermatological Properties of Its Active Fraction from the Whole Plant

**DOI:** 10.3390/molecules30020311

**Published:** 2025-01-14

**Authors:** Xing Wu, Meng-Fei Wanyan, Bao-Bao Shi, Rong Huang, Hui-Xiang Yang, Xian Wang, Ji-Kai Liu

**Affiliations:** 1 Anhui Province Key Laboratory of Bioactive Natural Products, School of Pharmacy, Anhui University of Chinese Medicine, Hefei 230012, China; wuxing812@outlook.com; 2 School of Pharmaceutical Sciences, South-Central Minzu University, Wuhan 430074, China; wymf666666@163.com (M.-F.W.); shibb0505@163.com (B.-B.S.); ronghuang@mail.scuec.edu.cn (R.H.); yanghuixiang@ahtcm.edu.cn (H.-X.Y.); xwang27@mail.scuec.edu.cn (X.W.)

**Keywords:** *Cuphea hookeriana*, cosmeceutical, tyrosinase, matrix metalloproteinase-1, skin whitening, dermatological

## Abstract

Natural products and botanicals continue to play a very important role in the development of cosmetics worldwide. The chemical constituents of a fine active fraction of the whole plant extract of *Cuphea hookeriana* Walp., and the tyrosinase and matrix metalloproteinase-1 (MMP-1) inhibitory and antioxidant activities of this fraction were investigated. The fine active fraction was mainly composed of seven natural compounds. The fine active fraction demonstrated substantial in vitro antioxidant potential using the ABTS assay (IC_50_ 1.66 μg/mL). It inhibited the two target enzymes (tyrosinase and MMP-1) engaged in skin whitening and aging with comparable IC_50_ values to the reference drugs. Acute toxicity experiments showed that mice gavage orally with the fine active fraction had no significant animal toxicity at a dose of 2000 mg/kg, and the maximum tolerated dose (MTD) in mice was greater than 2000 mg/kg. In a model where ultraviolet light promotes the increase in melanin secretion in guinea pig skin tissues, both α-arbutin and the fine active fraction can reduce melanogenesis, and the effect of the fine active fraction is better than that of α-arbutin.

## 1. Introduction

Aging, long-term exposure to unfavorable environments, malnutrition, fatigue, and other factors will lead to a decrease in the metabolic function of cells and skin tissues, a decrease in the blood circulation of the skin, a lack of moisture in the skin, an accumulation of structural and functional defects, endocrine disorders, and a decrease in the ability of the skin to repair itself, which will ultimately lead to a change in the skin’s appearance, physiological function, and texture. The most obvious changes in skin aging include the appearance of fine lines and wrinkles, loss of skin elasticity, skin sagging, laxity, loss of color, and discoloration [1].

Oxygen free radicals generated during cellular metabolism can cause cumulative damage to intracellular biomolecules, resulting in the loss of cellular senescence and proliferation, and this imbalance between oxidative and antioxidant systems is considered to be an important causative factor of skin aging [2]. Tyrosinase is a kind of oxidoreductase widely found in plants, animals, microorganisms, and the human body, and it is the key rate-limiting enzyme for melanin synthesis, which is closely related to the occurrence of excessive melanin deposition in the human body, such as freckles, brown spots, and other diseases. In recent years, its application in the fields of medicine, cosmetology, food, and environmental protection has attracted extensive attention at home and abroad. Most of the whitening agents in the market are based on the inhibition of tyrosinase to achieve a whitening effect [3].

The activation of matrix metalloproteinases (MMPs) in vivo, caused by external factors, can lead to the excessive degradation of collagen and elastin, which support the skin’s structure, resulting in symptoms of aging such as wrinkled, inelastic skin. During normal development, MMPs are responsible for the degradation of extracellular matrix (ECM) such as collagen, elastin, gelatin, matrix glycoproteins, and proteoglycans present in bone, cartilage, and skin. Among them, matrix metalloproteinase-1 (MMP-1), a calcium- and zinc-dependent vertebrate collagenase subclass, is a member of the MMP family capable of efficiently degrading type I and type III interstitial collagen, which is involved in a wide range of physiological and pathological processes, including tumors, cardiovascular diseases, multiple sclerosis, and skin aging. By inhibiting the expression or activity of MMP-1, the specific degradation of extracellular matrix components can be reduced. Therefore, eliminating excessive free radical damage during life activities and inhibiting collagenase activity, thereby reducing collagen loss by degradation, are believed to be effective in slowing down skin aging [4].

Reactive oxygen species (ROS) comprise non-radical and free radical species, which affect the epidermis and dermis. Free radicals produced in excess exert direct destructive effects on cell components. A high level of free radicals in the absence of effective antioxidant mechanisms may cause extensive damage to cellular structures [5]. Free radicals may damage the skin by destroying the lipid components of sebum and ceramides of the intercellular cement of the stratum corneum or by the oxidation of polyunsaturated fatty acids of cell membrane phospholipids [6], and directly damage the DNA and lipids of epidermal keratinocytes [7]. Moreover, ROS contributes to the oxidative stress (OxS)-induced degradation of melanocytes and compromises the function of cellular proteins, such as tyrosine-related protein 1 (TRP1) and tyrosinase, involved in melanogenesis [8]. ROS are also capable of inducing the expression of proteinases responsible for remodeling the extracellular matrix (ECM), such as serine proteases and matrix metalloproteinases (MMPs), mainly collagenase 1 (MMP-1).

With the growing consumer demand for cosmetic products used for whitening and antioxidant purposes and for slowing down the aging of the skin, efficacious cosmetic ingredients derived from plants have been widely used in the cosmetic industry [9]. These active ingredients come from various parts of plants, such as roots, stems, leaves, bark, seeds, fruits, healing tissues, protoplasts, and phloem tissues, and are incorporated into cosmetics in different forms, such as pure or semi-pure components, solid or liquid extracts, or derivatives. Plant cosmetics advocate for nature, mildness, and safety, and are highly popular among consumers, and creating plant-based cosmetics has become an inevitable trend in the development of the cosmetics industry [10].

In previous studies, the phytochemical characterization of the aqueous and ethanolic leaf extracts of *Cupressus arizonica* revealed 67 secondary metabolites belonging mainly to flavonoids, biflavonoids, phenolic acids, and proanthocyandins. The aqueous extract displayed substantial in vitro antioxidant activity and appreciable inhibitory activity against the four crucial enzymes (collagenase, elastase, tyrosinase, and hyaluronidase) which are involved in skin aging [11]. The active fraction of *Cynanchum atratum* effectively promotes skin whitening by reducing melanin production. The potential mechanisms include tyrosinase inhibition through restraining cAMP cascade and the MITF pathway, and augmenting antioxidant capacity to eliminate excessive oxidative stress [12].

The genus *Cuphea* (Lythraceae) is a monophyletic taxon comprising some 240–260 species that grow wild in the warm, temperate, and tropical regions of South and Central America and the southern part of North America. They have been valued as traditional medicinal remedies for numerous indications, including treating wounds, parasitic infections, hypertension, digestive disorders, cough, rheumatism, and pain. Modern pharmacological research provides data that support many of these traditional uses. Such a wide array of medicinal applications may be due to the exceptionally rich phytochemical profile of these plants, which includes bioactive compounds classified into various metabolite groups, such as polyphenols, triterpenes, alkaloids, and coumarins [13]. Seeds that exhibit intermediate storage behavior seem to die under conventional –18 °C storage conditions. *Cuphea wrightii*, *C. laminuligera*, *C. carthagenensis*., and *C. aequipetala* are considered sensitive to low-temperature storage. The seeds of these species have triacylglycerols (TAGs) that are crystalline at –18 °C and melt when the seeds are warmed to >35 °C. In contrast, the seeds of tolerant species, *C. lanceolata* and *C. hookeriana*, have TAG that crystallize at temperatures below –18 °C and are fluid at 22 °C [14]. *Cuphea hookeriana* Walp. is an annual or perennial evergreen plant in the family of Lythraceae. Its flowers are exquisite, and its long flowering period has a strong ornamental value and landscaping function. Now, Guangdong, Guangxi, Yunnan, Fujian, and other provinces in China have introduced its cultivation and it is widely used in gardening [15]. At present, there is no report on the antioxidant, whitening, and anti-aging for this plant [16].

This study was also intended to investigate the in vitro antioxidant and inhibitory activities of the *C. hookeriana* extracts or active fine fraction against the different skin key enzymes, mainly elastase, hyaluronidase, collagenase, and tyrosinase enzymes. It is hoped that this plant resource will be used in cosmetic development, especially for whitening purposes.

Arbutin is a compound of hydroquinone and D-glucose, and it has been over 30 years since there have been serious studies on the skin-lightening action of this substance. Studies on the antioxidant properties of arbutin are emerging, and these antioxidant properties are proposed to contribute to the skin-depigmenting action of arbutin [17]. In this study, it was selected as a positive control.

Kojic acid is a natural organic acid that inhibits tyrosinase by capturing copper ions in the active site of tyrosinase, thereby preventing melanin synthesis. In addition to this action, it also has antioxidant properties. Kojic acid has been found to be irritating at higher doses, and a topical concentration of 1.0% is currently recommended. It has been used at concentrations often ranging from 1 to 4%. Kojic acid has proven to be effective in the treatment of photodamage, hyperpigmented scars, and lentigines [9]. It was also used as another positive control.

## 2. Results and Discussion

### 2.1. Phytochemical Profiling by HPLC

**Figure 1:**
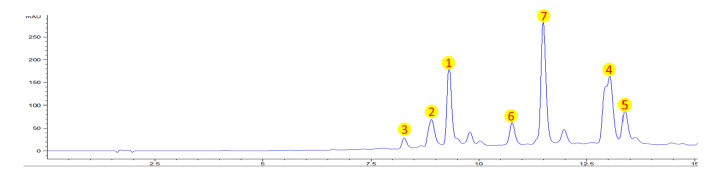
HPLC analytic profile of the active fine fraction from *C. hookeriana* whole plant (min).

**Figure 2:**
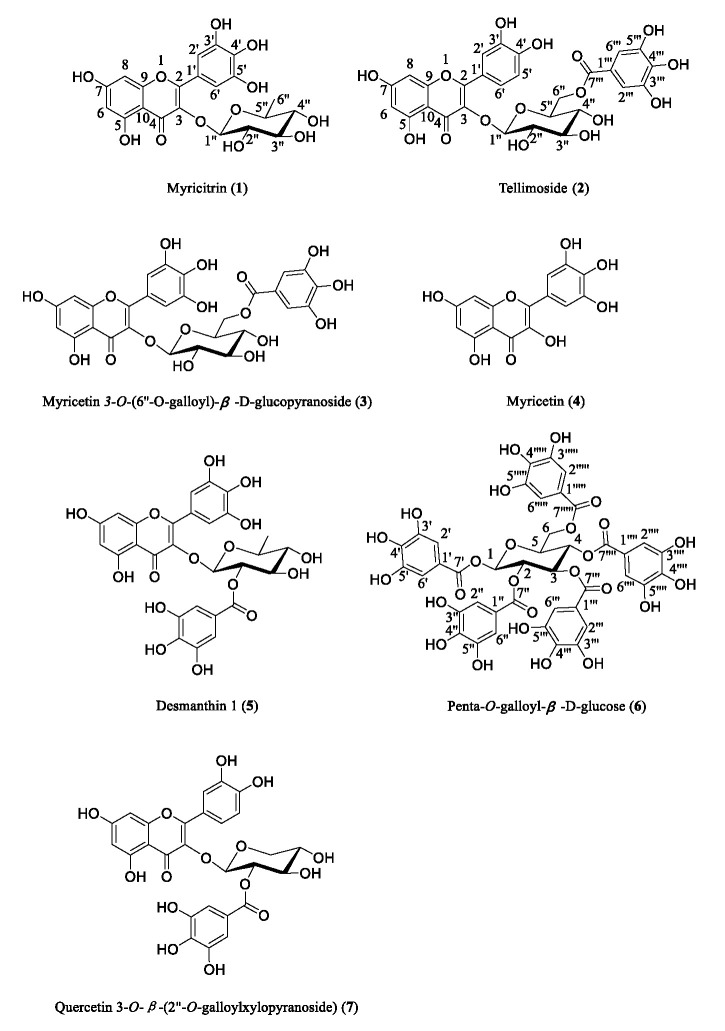
Structures of compounds **1**–**7**.

The crude ethanolic extract of *Cuphea hookeriana* whole plant was suspended in water, and partitioned with petroleum ether and EtOAc, respectively. The aqueous part was chromatographed and separated in a column with D101 resin by bioassay-guided fraction, and the active fine fraction (eluting with 50% methanol) was analyzed using HPLC (Figure 1). Altogether, seven main secondary metabolites belonging to the polyphenolic class were identified. The fine active fraction was mainly composed of seven natural compounds, which are myricitrin (**1**) [18], tellimoside (**2**) [19], myricetin 3-O-(6″-O-galloyl)-*β*-D-glucopyranoside (**3**) [20], myricetin (**4**) [21], desmanthin 1 (**5**) [22], penta-O-galloyl-*β*-D-glucose (**6**) [23], and quercetin 3-O-*β*-(2″-O-galloylxylopyranoside) (**7**) [24] (Figure 2).

Myricitrin, a botanical flavone, is abundantly distributed in the root bark of *Myrica cerifera*, *Myrica esculenta*, *Ampelopsis grossedentata*, *Nymphaea lotus*, *Chrysobalanus icaco*, and other plants. It was reported to possess effective anti-oxidative, anti-inflammatory, and anti-nociceptive activities [25]. Tellimoside was found to show free radical scavenging activity [26] and lipid peroxidation inhibiting activity [27]. Modern pharmacological studies showed that myricetin possesses a variety of biological activities such as anti-inflammatory, antitumor, antibacterial, antiviral, and anti-obesity effects, exerts cardiovascular protection, protects against neurological damage, and protects the liver against potential injuries [28]. The flavanone glucosides isolated from *M. multiflora* showed potent inhibitory activity on aldose reductase and α-glucosidase and among them, desmanthin-1 showed the most potent activity on aldose reductase [29]. Penta-*O*-galloyl-D-glucose (PGG) is a simple hydrolyzable tannin in plants. PGG exists in two anomeric forms, α-PGG and β-PGG. While β-PGG can be found in a wide variety of plants, α-PGG is rather rare in nature. Numerous studies with β-PGG revealed a wide variety of biological activities, such as anti-microbial and anti-cancer functions [30].

### 2.2. In Vitro Antioxidant Assay

**Figure 3:**
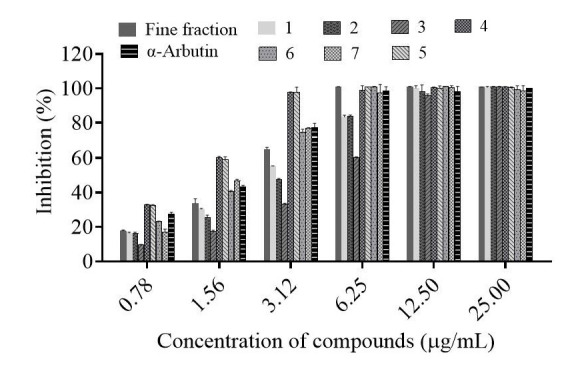
Scavenging effect of the fine fraction and compounds of *C. hookeriana* whole plant on ABTS free radicals (N = 3). Data are representative of three independent experiments analyzed by the nonlinear regression (curve fit) method with the GraphPad Prism10 software. Values are the mean ± SEM. Significant difference compared with a-arbutin. (fine fraction: *p* < 0.05; compounds **6**, **7**: *p* < 0.01 and compounds **1**–**5**: *p* < 0.001).

As shown in Figure 3, the fine fraction of *Cuphea hookeriana* and all seven compounds isolated compounds from this fine fraction showed good scavenging effects on ABTS radicals in the concentration range tested, with the fine fraction showing the best scavenging effects, and compounds **4** and **5** showing the best scavenging effects (Table 1). The antioxidant IC_50_ value of the fine fraction is 1.66 μg/mL. Meanwhile, the IC_50_ value of the positive control α-arbutin was 1.52 μg/mL. The above experimental results indicated that the fine fraction and compounds **1**–**7** possessed good antioxidant activity, and are comparable to those of α-arbutin.

### 2.3. Effect of Tyrosinase Inhibition

As shown in Table 2, the inhibition rates of tyrosinase by 0.3 mg/mL of the positive control kojic acid and α-arbutin were 97.9 ± 1.3% and 54.6 ± 2.8%, respectively. Among them, the fine fraction showed the best tyrosinase inhibition effect of 70.5 ± 1.9%, which was superior to the pure compounds **1**–**7**. There may be two reasons for this, one is that there may be a synergistic effect between the compounds in the active fine fraction; the other reason may be that there may be trace compounds with very high activity that we have not yet identified. The first is more likely.

**Figure 4:**
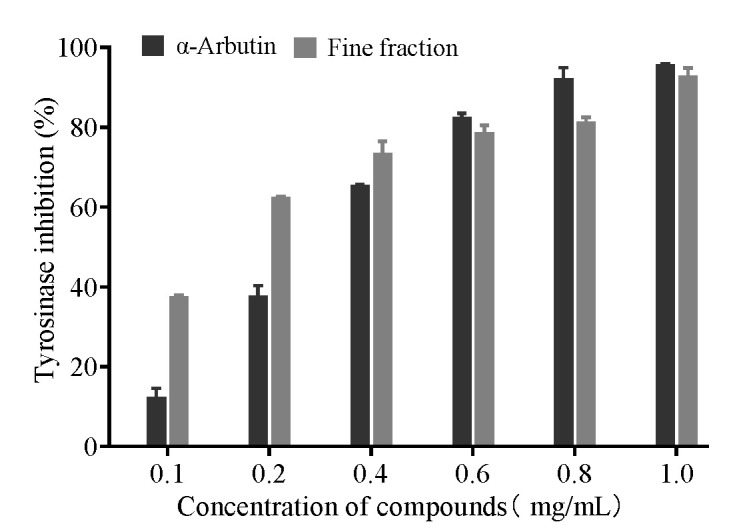
Inhibition of tyrosinase by the fine fraction of *C. hookeriana* and isolated compounds (N = 3). Data are representative of three independent experiments analyzed by the nonlinear regression (curve fit) method with the GraphPad Prism10 software. Values are the mean ± SEM. Significant difference between α-arbutin and the fine fraction (*p* < 0.01).

To further evaluate the in vitro inhibitory activity of the fine fraction on tyrosinase, the fraction and another positive control, α-arbutin, were assayed by diluting them with phosphate-buffered solution into different concentration gradients. As shown in Figure 4, both α-arbutin and the fine fraction exhibited a dose-dependent inhibition of tyrosinase activity, with the IC_50_s of 0.27 mg/mL and 0.14 mg/mL, respectively. The above experimental results indicated that the inhibitory effect of the fine fraction of *C. hookeriana* on tyrosinase was nearly twice as much as that of the positive control drug α-arbutin.

### 2.4. Effects of Matrix Metalloproteinase-1

**Figure 5:**
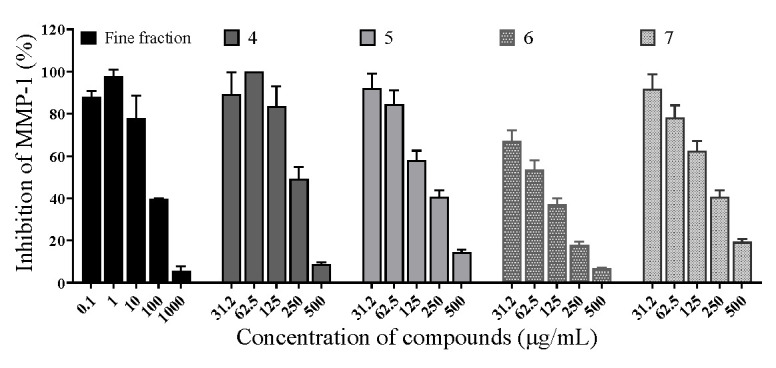
Inhibition of MMP-1 by the fine fraction of *C. hookeriana* and isolated compounds (N = 3). Data are representative of three independent experiments analyzed by the nonlinear regression (curve fit) method with the GraphPad Prism10 software. Values are the mean ± SEM. At a concentration of 100 μg/mL, the fine fraction inhibited matrix metalloproteinase-1 activity by 64% (*p* < 0.01 ).

The results are shown in Figure 5. Both the fine fraction of *C. hookeriana* and isolated compounds from this fraction exhibited a dose-dependent inhibition of matrix metalloproteinase-1 activities. At a concentration of 100 μg/mL, the fine fraction inhibited matrix metalloproteinase-1 activity by 64% (** *p* < 0.01). The IC_50_ of MMP-1 by the fine fraction of *C. hookeriana* was calculated as 55.0 μg/mL after applying the GraphPad Prism 8.0 software for nonlinear fitting; the IC_50_ of MMP-1 by compounds **4**, **5**, **6**, and **7** were 214.6, 155.4, 60.2, and 160.7 μg/mL, respectively. The above experimental results indicated that the fine fraction of *C. hookeriana* had a better inhibition effect on MMP-1.

### 2.5. Acute Toxicity Test in Mice

The mice were observed for 48 h after gavage of the higher dose of 2000 mg/kg. The mice showed good growth, normal activity, good fur color, and glossiness, and none of them showed symptoms of toxicity such as tremors, salivation, convulsions, stirrups, drowsiness, coma, etc. The mice were also found to be in good condition, with no obvious pathological changes. At the end of the experiment, a gross anatomical examination was carried out and no obvious pathological changes were observed in the organs. There was no significant difference in body weight between the blank group and the experimental group. Acute toxicity experiments showed that the fine fraction at a dose of 2000 mg/kg had no significant animal toxicity, and the maximum tolerated dose (MTD) in the mice was all greater than 2000 mg/kg.

### 2.6. UVB-Induced Skin Pigmentation in Guinea Pigs

**Figure 6:**
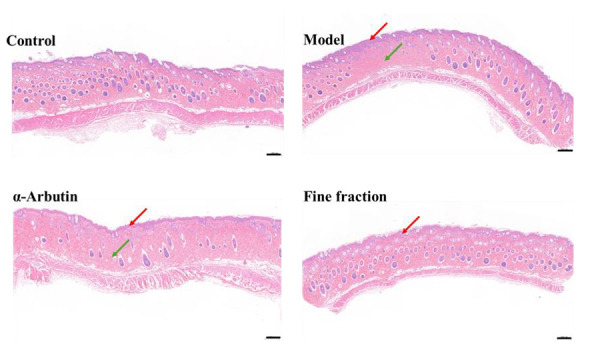
H&E staining of guinea pig dorsal skin tissue (The scale bar represents 500 μm. The red arrows indicate skin tissue thickening; the green arrows indicate a decrease in follicles).

HE staining can display the cellular and tissue morphology, but it cannot specifically identify melanin granules, because it is nonspecific [31]. The results of the H&E staining are shown in Figure 6; the epidermis of the skin tissue in the blank group was complete, the collagen fibers were arranged tightly, and the number of hair follicles was abundant. The skin tissue of the guinea pigs in the model group was obviously thickened; the dermis could be infiltrated with lymphocytes and the number of hair follicles was reduced. The skin tissue of the guinea pigs in the positive control group was epidermally thicker; the dermis was infiltrated by a large number of lymphocytes, the collagen fibers were sparsely arranged, and the number of hair follicles was less. The skin tissue of the guinea pigs in the experimental group had a thicker epidermis, with a large number of echinocytes with hydropic degeneration and sparse and light-stained cytoplasm; the dermis had a small number of granulocytes infiltrated, the collagen fibers were tightly arranged, and the number of hair follicles was abundant. The above results indicate that UV rays cause the thickening of guinea pig skin tissues, infiltration of inflammatory cells, damage of collagen fibers, and reduction in hair follicles. Compared with the positive control β-arbutin, the fine fraction inhibited skin tissue thickening, promoted collagen fiber proliferation, and promoted hair follicle proliferation.

**Figure 7:**
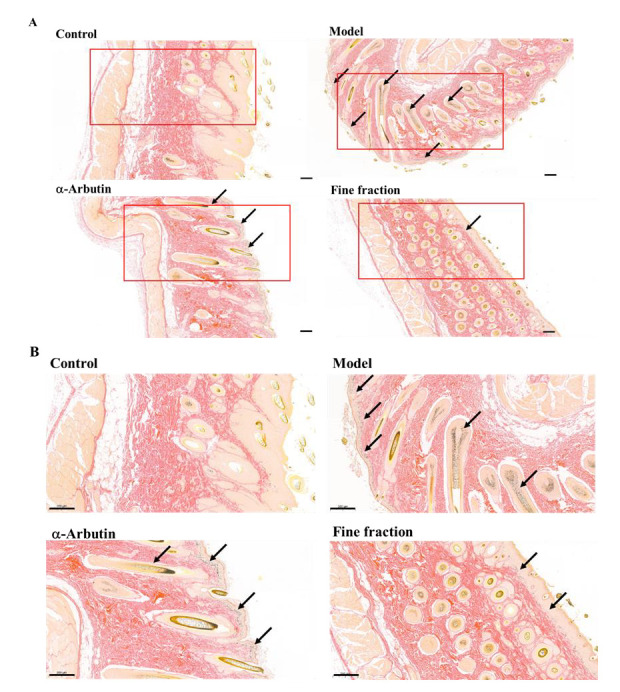
Fontana–Masson silver staining of guinea pig dorsal skin tissue (**A**). The scale bar represents 500 μm; (**B**). the scale bar represents 200 μm. The black arrows indicate melanin deposition. The red square in (**A**) represents (**B**).

The biopsy specimens were stained with Fontana–Masson which can enhance the visualization of melanin granules to assess the content of epidermal melanin after treatment with a-arbutin and fine fraction [31]. The results of melanin content detection are shown in Figure 7. The melanin content of the guinea pig back skin in the blank group control group was extremely small, mainly concentrated in the area of the basal layer, and only a very small amount of melanin existed in the spinous layer; the model group showed a significant increase in melanin after UV irradiation, and a large amount of melanin was expressed in the basal and spinous layers, but it was mainly concentrated in the basal layer; the positive control group contained more melanin, mainly concentrated in the basal layer and less in the spinous layer; the distribution of melanin in the experimental group was significantly reduced compared with the model group, mainly concentrated in the basal layer and less in the spinous layer. The above results show that ultraviolet rays promote the increase in melanin secretion in guinea pig skin tissues, and both β-arbutin and the fine fraction can reduce melanin production, and the effect of the fine fraction is better than that of α-arbutin, which exhibited a potential application as a whitening agent in cosmetics.

Oxidative stress is believed to be the main cause of skin aging and damage by a variety of mechanisms. Therefore, searching for plants that inhibit these mechanisms is an excellent way to slow down skin aging. Polyphenolic phytochemicals are well known for their potential antioxidant capacity. Therefore, plants containing antioxidants are promising candidates for preventing skin aging [16]. In the present study, the in vitro antioxidant and anti-aging properties of the active and fine fraction of the ethanolic extract of the whole plant of *C. hookeriana* were evaluated and their major constituent was isolated and identified.

We revealed that the water-soluble fraction of the ethanolic extract of the whole plant of *C. hookeriana* is a rich source of antioxidant compounds that can behave synergistically with skin aging target enzymes, thereby improving the skin aging process. Our results are in good agreement with the reported data on the anti-aging activity of some compounds.

At present, most of the skin-whitening and -brightening agents on the market are based on the inhibition of tyrosinase, such as α-arbutin, kojic acid, 4-butylresorcinol, etc., or based on antioxidants, such as vitamin C, vitamin E, and their derivatives, in order to achieve the effect of whitening [9]. α-Arbutin belongs to hydroquinone derivatives; it has a cytotoxic effect on melanocytes and strong skin irritation, and long-term use may cause permanent white spots on the skin, and it has been included in the list of banned ingredients in cosmetics by many countries [17]. Kojic acid inhibits the catalytic activity of tyrosinase by chelating its copper ions, and its use in commercialized skin and hair brighteners can lead to contact allergy and may cause skin lesions or liver cancer [9]. Vitamin C and vitamin E, as important antioxidants in the human body, do not directly inhibit tyrosinase, but reduce the colored intermediates in melanin biosynthesis, and have only an inadequate whitening and brightening effect on the skin. These unfavorable factors limit their wide application in the whitening and spot removal cosmetics market.

Our in vitro enzyme assays showed that the fine fraction of *C. hookeriana* had significant tyrosinase and matrix metalloproteinase-1 inhibitory activities and that the activity of the fine fraction was better than that of all seven compounds isolated from this fraction, suggesting that these compounds may have some synergistic effect. Our animal test results showed that UV light caused skin tissue thickening, inflammatory cell infiltration, collagen fiber damage, and hair follicle reduction in guinea pigs. Compared with the positive control α-arbutin, the fine fraction we used inhibited skin tissue thickening and promoted collagen fiber proliferation and hair follicle proliferation. In addition, ultraviolet light promoted the increase in melanin secretion in guinea pig skin tissues, and the fine active fraction reduced melanin production, and the effect of the fine active fraction was superior to that of the positive control α-arbutin.

## 3. Materials and Methods

### 3.1. General Experimental Procedures

D101 resin (Shanghai Macklin Biochemical Co., Ltd., Shanghai, China) and reversed-phase silica gel (Fuji Silysia Chemical Co., Kasugai, Japan, Lichroprep RP-18, 40–70 μm) were used for column chromatography (CC). Medium-pressure liquid chromatography (MPLC) was carried out on a Biotage (Uppsala, Sweden, C-615 Pump Manager, C-605 pump module, C-660 fraction collector, RP-18 column). The analytic HPLC was performed on Zorbax SB-C18 column (5 µm, 4.6 × 150 mm, gradient elution of acetonitrile-water 10–30%, *v*/*v*, 1.0 mL/min, 0–15 min) utilizing a DAD detector on an Agilent 1260 liquid chromatography system (Agilent Technologies, Santa Clara, CA, USA). The preparative HPLC was performed on a Zorbax SB-C18 column (5 µm, 9.4 × 150 mm) utilizing a DAD detector on an Agilent 1260 liquid chromatography system (Agilent Technologies, USA). The Bruker Avance III 600 MHz spectrometer (Bruker, Karlsruhe, Germany) was utilized to obtain the NMR spectra. On a Q Exactive Orbitrap mass spectrometer (Thermo Scientific, Waltham, MA, USA), the HRESIMS spectra were acquired.

### 3.2. Plant Material

*Cuphea hookeriana* Walp. was collected in Kunming, Yunnan Province of China, in October 2023. The plant material was identified by Prof. Dr. Hua Peng, Kunming Institute of Botany (Kunming, China), and a voucher specimen (No. HB202307) is held at South-Central Minzu University.

### 3.3. Extraction and Isolation

The dried and crushed materials of the whole plant *Cuphea hookeriana* (25 kg) were extracted with 95% ethanol at room temperature for two weeks, with maceration performed three times. The solvent was evaporated under reduced pressure to yield the EtOH extract, and this extract was suspended in water and extracted with petroleum ether and EtOAc, respectively.

The aqueous fraction was chromatographed in a column with 5 kg of D101 resin. The pretreated resin was first filled into the column by wet loading. The column was pressed with distilled water until the height of the column remained unchanged, and then the aqueous sample was slowly added along the wall of the column. After all the samples were added to the column, when the liquid level dropped to about 5 mm above the column surface, a small amount of distilled water was used to rinse the wall of the column, and the liquid on the column surface was clarified and colorless for 2–3 times, and then the column was slowly filled with distilled water, and the outlet port was closed, and the column was allowed to stand overnight (12 h) until adsorption equilibrium was reached. The chromatographic column was eluted sequentially with 30 L of methanol/water solution (*v*/*v*) at concentrations of 0%, 50%, and 100% at a flow rate of 100 mL/min. The components were concentrated under reduced pressure at 50 °C to obtain 650 g of 0% methanol solution (*v*/*v*) fraction, 135 g of 50% methanol solution (*v*/*v*) fraction, and 142 g of 100% methanol solution (*v*/*v*) fraction.

The 50% methanol eluting fraction (135 g) collected by column chromatography was further purified by hydroxypropyl dextran gel column chromatography, and eluted by methanol/water (70:30, *v*/*v*) and chloroform/methanol (50:50, *v*/*v*) solutions. The eluted portion of the methanol/water (70:30, *v*/*v*) solution was the active ingredient.

The methanol/water (70:30, *v*/*v*) eluting fraction (75 g) collected was further purified by RP18 column chromatography. An appropriate amount of methanol was added to the fraction (75 g) as solvent to dissolve and transfer to an evaporating dish, and 100 g of reversed-phase silica gel (Fuji Silysia Chemical Co., Japan, Lichroprep RP-18, 40–70 μm) was added to adsorb the samples, and the samples were air-dried and grounded into powder, followed by the use of medium-pressure chromatography, room temperature, flow rate of 25 mL/min, pressure: 15 MPa, and water/methanol system gradient elution (0:100, 20:80, 40:60, 60:40, 80:20, and 100:0; *v*/*v*). The eluent was concentrated and recovered after eluting 5 L of each ratio. The methanol/water (20:80 and 40:60, *v*/*v*) eluted fraction was collected as *Cuphea hookeriane* fine fraction (45 g).

A total of 120 mg of the fine fraction of *Cuphea hookeriane* was prepared by high-performance liquid chromatography (column: Agilent Zorbax SB-C18 column, size: 9.4 mm × 250 mm; gradient elution of acetonitrile-water, 10–30%, *v*/*v*; 4 mL/min), concentrated and dried in vacuum to obtain compound **1** (yellow powder, 10.5 mg, tR = 33.5 min), compound **2** (yellow powder, 8.8 mg, tR = 32.6 min), compound **3** (yellow powder, 7.7 mg, tR = 30.4 min), compound **4** (yellow powder, 22.5 mg, tR = 44.6 min), compound **5** (yellow powder, 8.9 mg, tR = 46.3 min), compound **6** (yellow powder, 9.4 mg, tR = 36.2 min), and compound **7** (yellow powder, 10.5 mg, tR = 38.5 min).

*Myricitrin* (**1**): yellow powders, ^1^H NMR (600 MHz, CD_3_OD, *δ*, ppm, *J*/Hz): 6.95 (s, H-2′/H-6′), 6.36 (d, *J* = 2.1, H-8), 6.20 (d, *J* = 2.1, H-6), 5.32 (d, *J* = 1.6, H-1”), 4.22 (dd, *J* = 3.4, 1.6, H-2″), 3.79 (dd, *J* = 9.6, 3.4, H-3″), 3.34 (t, *J* = 9.6, H-4″), 3.52 (dd, *J* = 9.6, 6.2, H-5″), 0.96 (d, *J* = 6.2, H-6″). ^13^C NMR (150 MHz, *δ*, ppm): 178.3 (C-4), 164.5 (C-7), 161.7 (C-5), 158.0 (C-2), 157.1(C-9), 145.5 (C-3′/5′), 136.5 (C-4′), 134.9 (C-3), 120.5 (C-1′), 108.2 (C-2′/6′), 104.5 (C-10), 102.2 (C-1″), 98.4 (C-6), 93.3 (C-8), 71.9 (C-4″), 70.7 (C-3″), 70.6 (C-5″), 70.5 (C-2″), 16.3 (C-6″) [18].
*Tellimoside* (**2**): yellow powders, ^1^H NMR (600 MHz, CD_3_OD, *δ*, ppm, *J*/Hz): 7.78 (d, *J* = 2.2, H-2′), 7.56 (dd, *J* = 8.4, 2.2, H-6′), 6.89 (s, H-2″′/6″′), 6.81 (d, *J* = 8.4, H-5′), 6.37 (d, *J* = 2.0, H-8), 6.17 (d, *J* = 2.0, H-6), 5.10 (d, *J* = 7.7, H-1″), 4.32 (dd, *J* = 11.0, 6.9, H-6″), 4.20 (dd, *J* = 11.0, 6.0, H-6″), 3.88 (d, *J* = 3.7, H-4″), 3.85 (m, dd, *J* = 9.7, 7.7 H-2″), 3.80 (dd, *J* = 6.9, 6.0 H-5″), 3.60 (dd, *J* = 9.7, 3.7, H-3″). ^13^C NMR (150 MHz, *δ*, ppm): 178.1 (C-4), 166.6 (C-7′″), 164.6 (C-7), 161.4 (C-5), 157.6(C-2), 156.9 (C-9), 148.5 (C-4′), 144.9 (C-3′″/5′″), 144.3 (C-3′), 138.4 (C-4′″), 134.3 (C-3), 121.6 (C-1′), 121.4 (C-6′), 119.6 (C-1′″), 116.4 (C-2′), 114.7 (C-5′), 108.7 (C-2′″/6′″), 104.1 (C-10), 104.1 (C-1″), 98.6 (C-6), 93.4 (C-8), 73.6 (C-5″), 73.1 (C-3″), 71.6 (C-2″), 68.6 (C-4″), 62.3 (C-6″) [19].
*Myricetin 3-O-(6″-O-galloyl)-β-D-glucopyranoside* (**3**): yellow powders, ^1^H NMR (600 MHz, CD_3_OD, *δ*, ppm, *J*/Hz): 7.27 (s, H-2′/H-6′), 7.14 (s, H-2″′/6″′), 6.32 (d, *J* = 2.2, H-6), 6.15 (d, *J* = 2.2, H-8), 5.78 (d, *J* = 7.9, H-1”), 5.45 (dd, *J* = 9.9, 7.9, H-2”), 3.96 (dd, *J* = 3.5, 1.2, H-4”),3.85 (dd, *J* = 9.9, 3.5, H-3”), 3.70 (d, *J* = 6.1, H-6”), 3.61 (td, *J* = 6.1, 1.2, H-5′). ^13^C NMR (150 MHz, *δ*, ppm): 177.5 (C-4), 166.9 (C-7′″), 164.3 (C-7), 161.7 (C-9), 156.8 (C-5), 156.6 (C-2), 144.9 (C-3′/5′), 144.8 (C-3′″/5′″), 138.4 (C-4′″), 136.5 (C-4′), 133.9 (C-3), 120.6 (C-1′), 120.2 (C-1′″), 109.2 (C-2′″/6′″), 108.4 (C-2′/6′), 104.5 (C-10), 99.9 (C-1″), 98.2 (C-8), 93.1 (C-6), 76.1 (C-5″), 73.2 (C-2″), 72.1 (C-3″), 69.1 (C-4″), 60.6 (C-6″) [20].
*Myricetin* (**4**): yellow powders, ^1^H NMR (600 MHz, CD_3_OD, *δ*, ppm, *J*/Hz): 7.34 (s, H-2′/H-6′), 6.38 (d, *J* = 2.2, H-6), 6.18 (d, *J* = 2.2, H-8). ^13^C NMR (150 MHz, *δ*, ppm): 175.9 (C-4), 164.2 (C-7), 161.1 (C-5), 156.8 (C-9), 146.6 (C-2), 145.3 (C-3′/5′), 136.0 (C-3), 135.5 (C-4′), 121.7 (C-1′), 107.1 (C-2′/6′), 103.1 (C-10), 97.8 (C-8), 93.0 (C-6) [21].
*Desmanthin 1* (**5**): yellow powders, ^1^H NMR (600 MHz, CD_3_OD, *δ*, ppm, *J*/Hz): 7.08 (s, H-2″′/6″′), 6.98 (s, H-2′/H-6′), 6.36 (d, *J* = 2.2, H-6), 6.19 (d, *J* = 2.2, H-8), 5.63 (dd, *J* = 3.4, 1.8 H-2”), 5.51 (d, *J* = 1.8, H-1”), 4.05 (dd, *J* = 9.1, 3.4, H-3”),3.52 (dq, *J* = 9.1, 5.8, H-5”), 3.48 (t, *J* = 9.1, H-4”), 1.04 (d, *J* = 5.8, H-6”). ^13^C NMR (150 MHz, *δ*, ppm): 177.9 (C-4), 166.1 (C-7′″), 164.5 (C-7), 161.8 (C-9), 158.1 (C-2), 157.1 (C-5), 145.5 (C-3′/5′), 145.1 (C-3′″/5′″), 138.5 (C-4′″), 136.6 (C-4′), 134.2 (C-3), 120.4 (C-1′), 119.8 (C-1′″), 108.9 (C-2′″/6′″, 108.2 (C-2′/6′), 104.5 (C-10), 99.1 (C-1″), 98.2 (C-8), 93.3 (C-6), 72.5 (C-4″), 72.1 (C-2″), 70.8 (C-5″), 39.6 (C-3″), 16.4 (C-6″) [22].
*Penta-O-galloyl-β-D-glucose* (**6**): yellow powders, ^1^H NMR (600 MHz, CD_3_OD, *δ*, ppm, *J*/Hz): 7.02, 6.96, 6.88, 6.86, 6.80 (5×2′/6′), 6.14 (d, *J* = 8.3 Hz, H-1), 5.81 (t, *J* = 9.9 Hz, H-3), 5.51 (t, *J* = 9.9 Hz, H-4), 5.48 (dd, *J* = 9.9, 8.3 Hz, H-2), 4.41 (dd, *J* = 12.1, 1.9 Hz, H-6), 4.32 (dd, *J* = 4.3, 1.9 Hz, H-5), 4.28 (dd, J = 12.1, 4.3 Hz, H-6). ^13^C NMR (150 MHz, *δ*, ppm): 166.6, 165.9, 165.6, 165.5, 164.8 (5×C-7′), 145.2, 145.1, 145.1, 145.0, 144.9 (5×C-3′/5′), 139.4, 139.0, 138.9, 138.7, 138.6 (5×C-4′), 119.7, 119.0, 118.9, 118.8, 118.3 (5×C-1′), 109.3, 109.1, 109.1, 109.0, 109.0 (5×C-2′/6′), 92.4 (C-1), 73.0 (C-5), 72.7 (C-3), 70.8 (C-2), 68.4 (C-4), 61.8 (C-6) [23].
*Quercetin 3-O-β-(2”-O-galloylxylopyranoside)* (**7**): yellow powders, ^1^H NMR (600 MHz, CD_3_OD, *δ*, ppm, *J*/Hz): 7.61 (d, *J* = 2.3 Hz, H-2′), 7.53 (dd, *J* = 8.4, 2.3 Hz, H-6′), 7.13 (s, H-2′″, H-6′″), 6.84 (d, *J* = 8.4 Hz, H-5′), 6.18 (d, *J* = 2.2 Hz, H-6), 6.36 (d, *J* = 2.2 Hz, H-8), 5.55 (d, *J* = 5.9 Hz, H-1″), 5.46 (dd, *J* = 7.6, 5.9 Hz, H-2″), 3.50 (m, H-4″), 3.89 (m, H-5″). ^13^C NMR (150 MHz, *δ*, ppm): 177.8 (C-4), 166.3 (C-7′″), 164.4 (C-7), 161.7 (C-5), 157.1 (C-2), 156.9 (C-9), 148.4 (C-4′, 145.0 (C-3′″/5′″), 144.7 (C-3′), 138.6 (C-4′″), 133.7 (C-3), 121.9 (C-1′), 121.5 (C-6′), 120.0 (C-1′″), 115.7 (C-2′), 114.9 (C-5′), 109.1 (C-2/6′″), 104.4 (C-10), 99.5 (C-6), 98.4 (C-1″), 93.2 (C-8), 72.5 (C-2″), 70.4 (C-3″), 67.5 (C-4″), 64.8 (C-5″) [24].

### 3.4. In Vitro Colorimetric Assay

The antioxidant activity was estimated using the ABTS assay following the previously reported protocol [32].

### 3.5. Tyrosinase Inhibitory Assay

With slight modifications, the tyrosinase inhibitory activity was determined using the method of Song et al. [33]. In this investigation, *L-*dopa was used as the substrate, and mushroom tyrosinase was used. Different concentrations of the samples were dissolved in dimethyl sulfoxide (DMSO). Phosphate buffer (0.2 M, pH = 6.8), *L*-dopa solution (2 mM), and tyrosinase solution (100 U/mL) were prepared separately. The 200 μL reaction system was prepared according to Table 3. The reaction system was incubated at 37 °C in a microplate thermostatic shaker for 30 min, and then the absorbance at 475 nm was measured in a multifunctional enzyme labeling instrument (SPARK 10M, TECAN, Männedorf, Switzerland). Kojic acid and α-arbutin were used as the positive controls. Tyrosinase inhibition was calculated by the following formula:

Tyrosinase inhibition (%) = [1 − (A1 − A2)/(B1 − B2)] × 100%(1)

Sample group A1, sample negative control group A2, enzyme standard group B1, and enzyme negative control group B2.

### 3.6. Matrix Metalloproteinase-1 Inhibitory Assay

With slight modifications, the tyrosinase inhibitory activity was determined using the method of Amer et al. [34]. The 100 μL reaction system was prepared according to Table 4. MMP-1 enzyme stock solution was sequentially added into black 96-well plates to test the inhibitory effect of each sample on the MMP-1 enzyme, and three parallels were set for each sample concentration. The reaction system was shaken and mixed well, and the substrate was added after 5 min of warm bath at 37 °C. The initial fluorescence signal F0 of each well and the fluorescence signal F1 of the samples after 2.5 h of reaction were then measured by a multifunctional enzyme marker (SPARK 10M, TECAN), and the relative enzyme activities of the samples were calculated according to the following equations, in which the initial fluorescence signal of the enzyme control was set to be F0e, and that of the samples after 2.5 h of reaction was set to be F1e. The initial fluorescence signal of each sample group was set as F0s, and the fluorescence signal of the sample solution after the 2.5 h reaction was set as F1s.

Matrix metalloproteinase-1 relative activity (%) = (F1s − F0s)/(F1e − F0e) × 100%(2)

### 3.7. Acute Toxicity Test in Mice

Healthy Kunming mice weighing 18–22 g were purchased from Liaoning Changsheng Biotechnology Co. (Shenyang, China). All the mice were housed in an environment with a temperature of 25 °C ± 2 °C and a relative humidity of 55% ± 15%, with 12 h/12 h alternating light and dark. All the mice were allowed to consume water and feed freely and were acclimatized for 7 days. All the procedures were approved by the Animal Ethics Committee of South-Central Minzu University. Using a previously published method [35], the mice were given saline, 25% DMSO solution (solvent control), and the fine fraction of *Cuphea hookeriana* by oral gavage, respectively. After oral gavage, the mice were observed for 2 days using a variety of indicators, including appearance, behavior, secretions, and excretions. The acute toxicity of the fine fraction of *Cuphea hookeriana* was expressed as a 50% lethal dose (LD_50_), which was calculated using the AOT425 statistical program.

### 3.8. UVB-Induced Skin Pigmentation in Guinea Pigs

Twenty guinea pigs, female, weighing 200–250 g, with large brownish-yellow hairs on the back, were purchased from Liaoning Changsheng Biotechnology Co. All the guinea pigs were housed in an environment with a temperature of 25 °C ± 2 °C and a relative humidity of 55% ± 15%, with 12 h/12 h alternating light and dark. All the guinea pigs were allowed to consume water and guinea pig-specific feed freely and were acclimatized to feeding for 7 days. This study was approved by the Animal Ethics Committee of South-Central Minzu University.

After one week of acclimatization, the guinea pigs were selected to have their brown hair areas depilated so that the back of the guinea pigs exposed 3 × 3 cm^2^ of skin. After depilation, the guinea pig’s back skin was irradiated with a 20 W UV lamp (Philips TL/2 lamp, Signify, Eindhoven, The Netherlands, 320 nm UVB) as the irradiation light source at a distance of 20 cm from the skin height. The UV intensity was 0.3–0.6 J/cm^2^ [36,37]. The control group was applied with arbutin (1%) and the treatment group was applied with the fine fraction of *Cuphea hookeriana* (2%) dissolved in 0.3% CMC-Na. The hyperpigmented areas were treated once a day for 20 days. At the end of the experiment, the guinea pigs were euthanized, and the dorsal skin tissues were removed and fixed in 4% paraformaldehyde fixative for the subsequent experiments.

### 3.9. Histopathological Examination

The excised guinea pig tissues were fixed in 4% paraformaldehyde and embedded in paraffin. The skin specimens were then sectioned at a thickness of 5 μm, stained with hematoxylin and eosin (H&E), and the histologic properties of the skin were observed under a light microscope.

### 3.10. Fontana–Masson Staining

To analyze the changes in the melanin content of the skin tissues, the guinea pig skin was taken and fixed in 4% paraformaldehyde for 24 h and paraffin-embedded. The sections were stained with Fontana–Masson. The stained slides were examined under a light microscope.

### 3.11. Statistical Analyses

All the data were expressed in at least three independent experiments. Multiple comparisons were analyzed using a one-way analysis of variance (ANOVA), and *p* < 0.05 was considered to be statistically significant.

## 4. Conclusions

The chemical constituents of a fine fraction of the whole plant extract of *Cuphea hookeriana* Walp., and the tyrosinase and matrix metalloproteinase-1 (MMP-1) inhibitory and antioxidant activities of this fraction were investigated. The fine active fraction was mainly composed of seven natural compounds, which are myricitrin (**1**), tellimoside (**2**), myricetin 3-O-(6″-O-galloyl)-*β*-D-glucopyranoside (**3**), myricetin (**4**), desmanthin 1 (**5**), penta-O-galloyl-*β*-D-glucose (**6**), and quercetin 3-O-*β*-(2″-O-galloylxylopyranoside) (**7**). The fine fraction demonstrated substantial in vitro antioxidant potential using the ABTS assay (IC_50_ 1.66 μg/mL). It inhibited the two target enzymes (tyrosinase and MMP-1) engaged in skin whitening and aging with comparable IC_50_ values to the reference drugs. In a model where ultraviolet light promotes the increase in melanin secretion in guinea pig skin tissues, both α-arbutin and the fine fraction can reduce melanogenesis, and the effect of the fine fraction is better than that of α-arbutin. Moreover, the manufacturing cost of the fine fraction is much lower, and it has more advantages than the pure compounds both in terms of effect and price cost when this plant resource is used for cosmeceutical applications.

In summary, the active fraction of *Cuphea hookeriana* effectively promotes skin whitening by reducing melanin production. The potential mechanisms include tyrosinase inhibition and augmenting antioxidant capacity to eliminate excessive oxidative stress. Hence, these findings will be a valuable contribution to the development of functional agents for skin issues. It is particularly noteworthy that the plant is widely cultivated as a horticultural plant in China, and the raw material is readily available from a very rich source. The main components in the active fraction have been identified; therefore, quality control is easy. Animal toxicity tests have shown that it is very safe. In summary, the plant has all the conditions to be used as a skin cosmetic of natural origin and is worthy of further development and utilization.

## Figures and Tables

**Table 1:** In vitro antioxidant activities (ABTS) for the fine fraction of *C. hookeriana* (μg/mL, N = 3).

Sample	IC_50_	Sample	IC_50_
Fine fraction	1.66	**4**	1.15
**1**	2.52	**5**	1.16
**2**	2.98	**6**	1.73
**3**	4.42	**7**	1.68
		α-arbutin	1.52

ABTS: 2,2′-Azinobis-(3-ethylbenzthiazoline-6-sulphonate).

**Table 2:** In vitro tyrosinase inhibitory activities for the fine fraction of *C. hookeriana* (%, N = 3).

Samples.	Inhibition Rate	Samples	Inhibition Rate
Fine fraction	70.5 ± 1.9	**4**	66.0 ± 4.9
**1**	52.5 ± 0.5	**5**	49.8 ± 7.6
**2**	50.9 ± 0.7	**6**	63.1 ± 2.1
**3**	61.4 ± 5.2	**7**	47.1 ± 1.4
α-arbutin	54.6 ± 2.	Kojic acid	97.9 ± 1.3

**Table 3:** Reaction system formulation for tyrosinase activity inhibition assay (volume/mL).

	Samples	A_1_	A_2_	B_1_	B_2_
Reagents					
Phosphate-buffered solution		20	140	40	160
Tyrosinase solution		40	40	40	40
Sample solution		20	20	0	0
L-Dopa		120	0	120	0

**Table 4:** Reaction system formulation for determination of matrix metalloproteinase-1 activity (mL/well).

	Enzyme Control	Negative Control	Samples
MMP-1 stock solution	65	0	65
Sample solution	—	—	10
Test buffer	10	75	—
FRET substrate	25	25	25

## Data Availability

The data presented in this study are available in the manuscript and Supplementary Materials.

## Supplementary Materials

The following supporting information can be downloaded at https://www.mdpi.com/article/10.3390/molecules30020311/s1, Figures S1–S14: The HRESIMS and NMR spectrum of compounds **1**–**9**.
