# Supplementary material for: Cuphea hookeriana: Phytochemical Profile and the Cosmeceutical and Dermatological Properties of Its Active Fraction from the Whole Plant
Source: Molecules. 2025 Jan 14;30(2):311. doi: 10.3390/molecules30020311 (PMC11767586; doi:10.3390/molecules30020311)
Supplement: Supplementary file 1 [file molecules-30-00311-s001.zip › molecules-3392844-supplementary.pdf]

SUPPLEMENTARY MATERIAL

Cuphea hookeriana: Phytochemical Profile and the Cosmeceutical and Dermatological Properties of Its Active Fraction from the Whole Plant

Xing Wu [1,2], Meng-Fei Wanyan [2], Bao-Bao Shi [2], Rong Huang [2], Hui-Xiang Yang [2], Xian Wang [2] and Ji-Kai Liu [1,2,*]

[1] Anhui Province Key Laboratory of Bioactive Natural Products, School of Pharmacy, Anhui University of Chinese Medicine, Hefei 230012, China; wuxing812@outlook.com

[2] School of Pharmaceutical Sciences, South-Central Minzu University, Wuhan 430074, China; wymf666666@163.com (M.-F.W.);shibb0505@163.com (B.-B.S.); ronghuang@mail.scuec.edu.cn (R.H.); yanghuixiang@ahtcm.edu.cn (H.-X.Y.); xwang27@mail.scuec.edu.cn (X.W.)

* Correspondence: liujikai@ahtcm.edu.cn

Contents

Figure S1 ^{1}H NMR spectrum of **1**. 3

Figure S2 ^{13}C NMR spectrum of **1.** 4

Figure S3 ^{1}H NMR spectrum of **2**. 5

Figure S4 ^{13}C NMR spectrum of **2.** 6

Figure S5 ^{1}H NMR spectrum of **3**. 7

Figure S6 ^{13}C NMR spectrum of **3.** 8

Figure S7 ^{1}H NMR spectrum of **4**. 9

Figure S8 ^{13}C NMR spectrum of **4.** 10

Figure S9 ^{1}H NMR spectrum of **5**. 11

Figure S10 ^{13}C NMR spectrum of **5.** 12

Figure S11 ^{1}H NMR spectrum of **6**. 13

Figure S12 ^{13}C NMR spectrum of **6.** 14

Figure S13 ^{1}H NMR spectrum of **7**. 15

Figure S14 ^{13}C NMR spectrum of **7.** 16

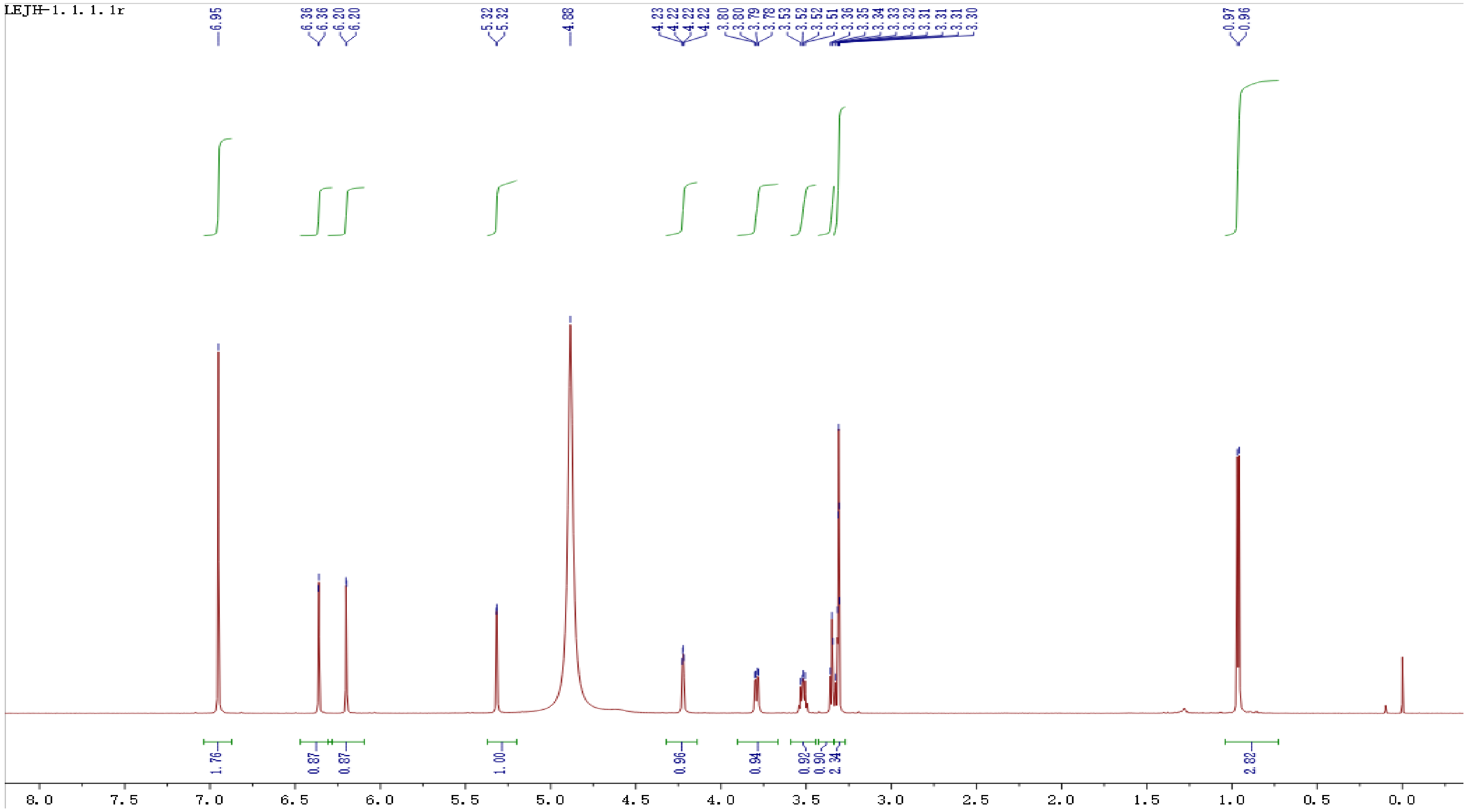

Figure S1 ^{1}H NMR spectrum of **1**.

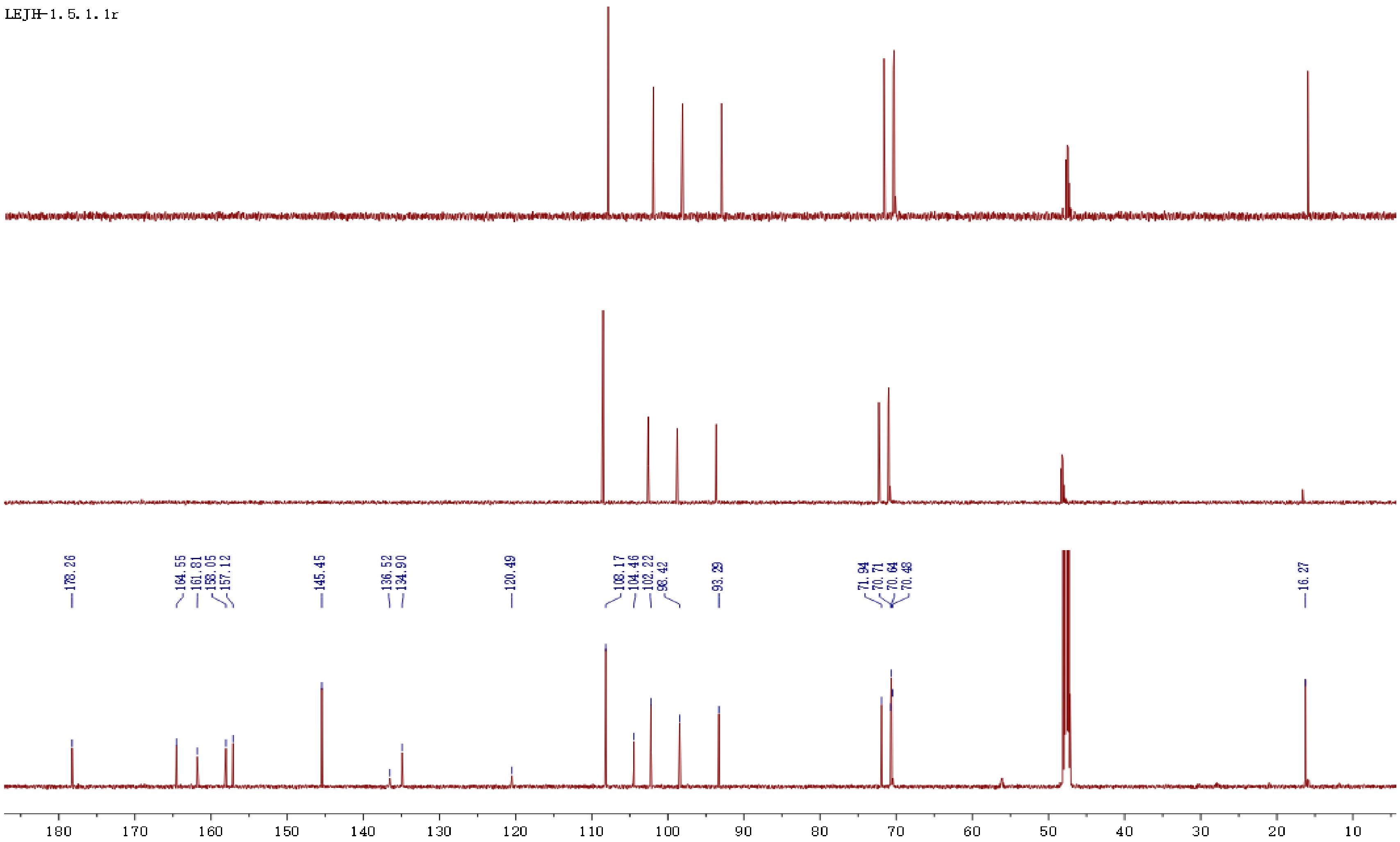

Figure S2 ^{13}C NMR spectrum of **1.**

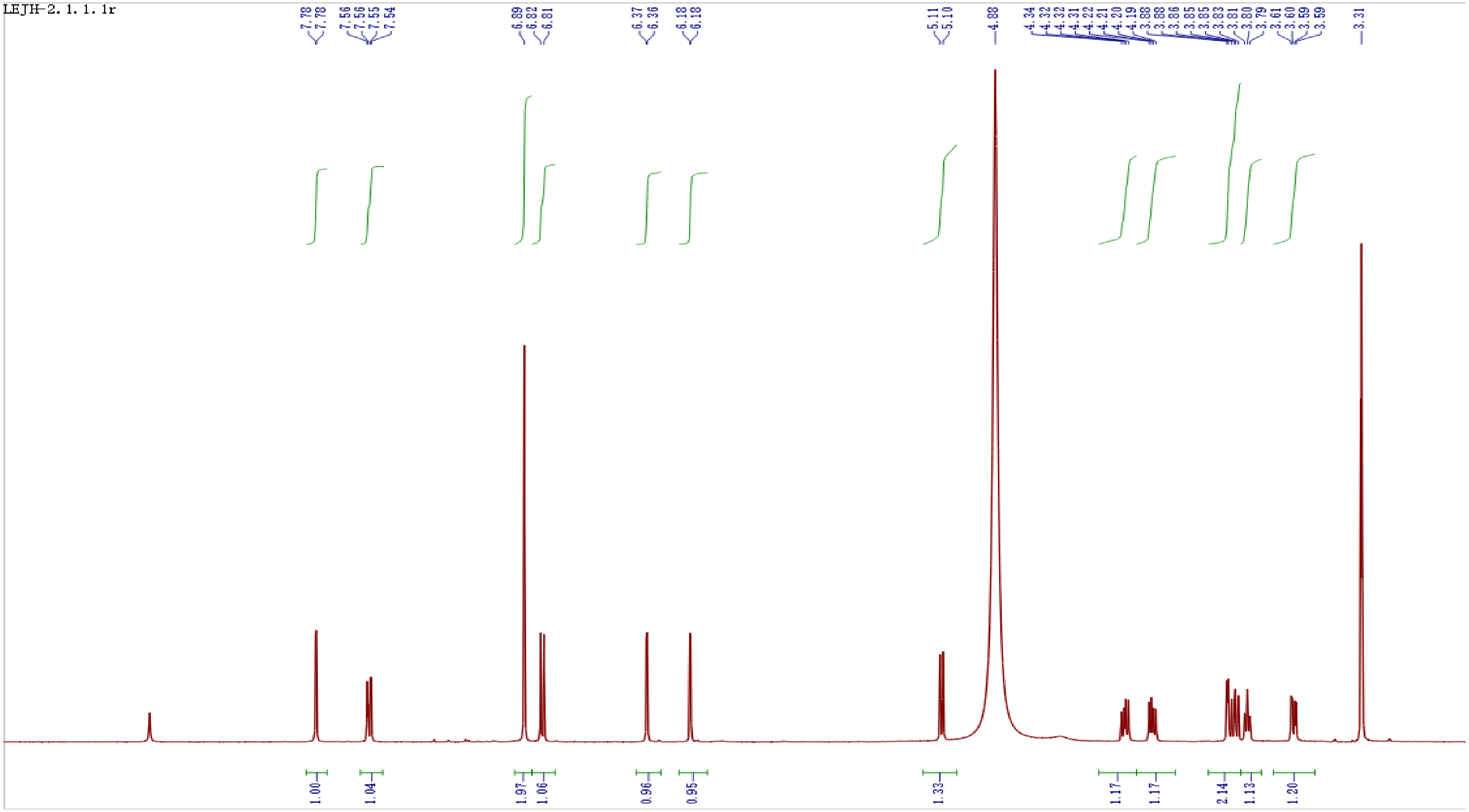

Figure S3 ^{1}H NMR spectrum of **2**.

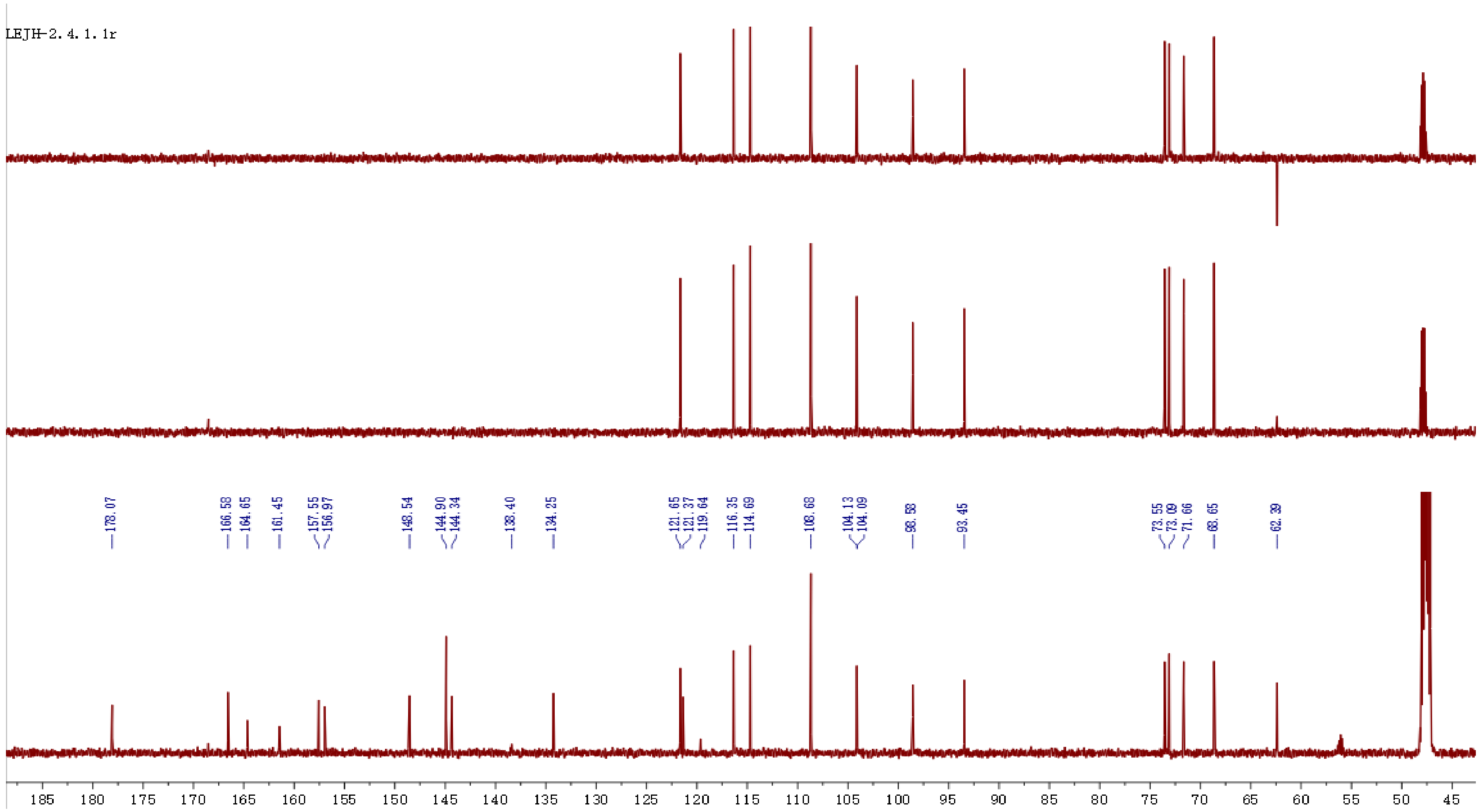

Figure S4 ^{13}C NMR spectrum of **2.**

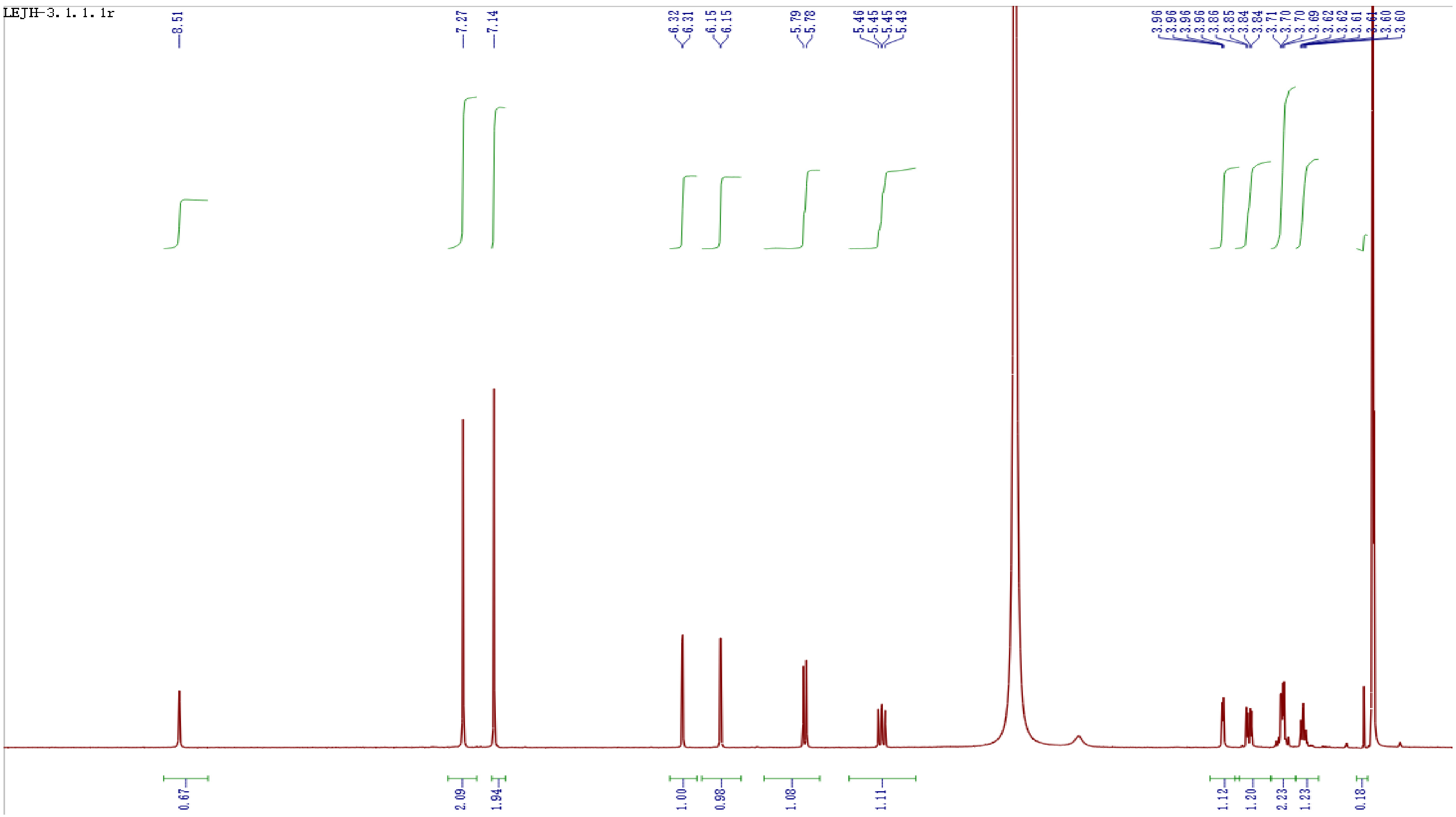

Figure S5 ^{1}H NMR spectrum of **3**.

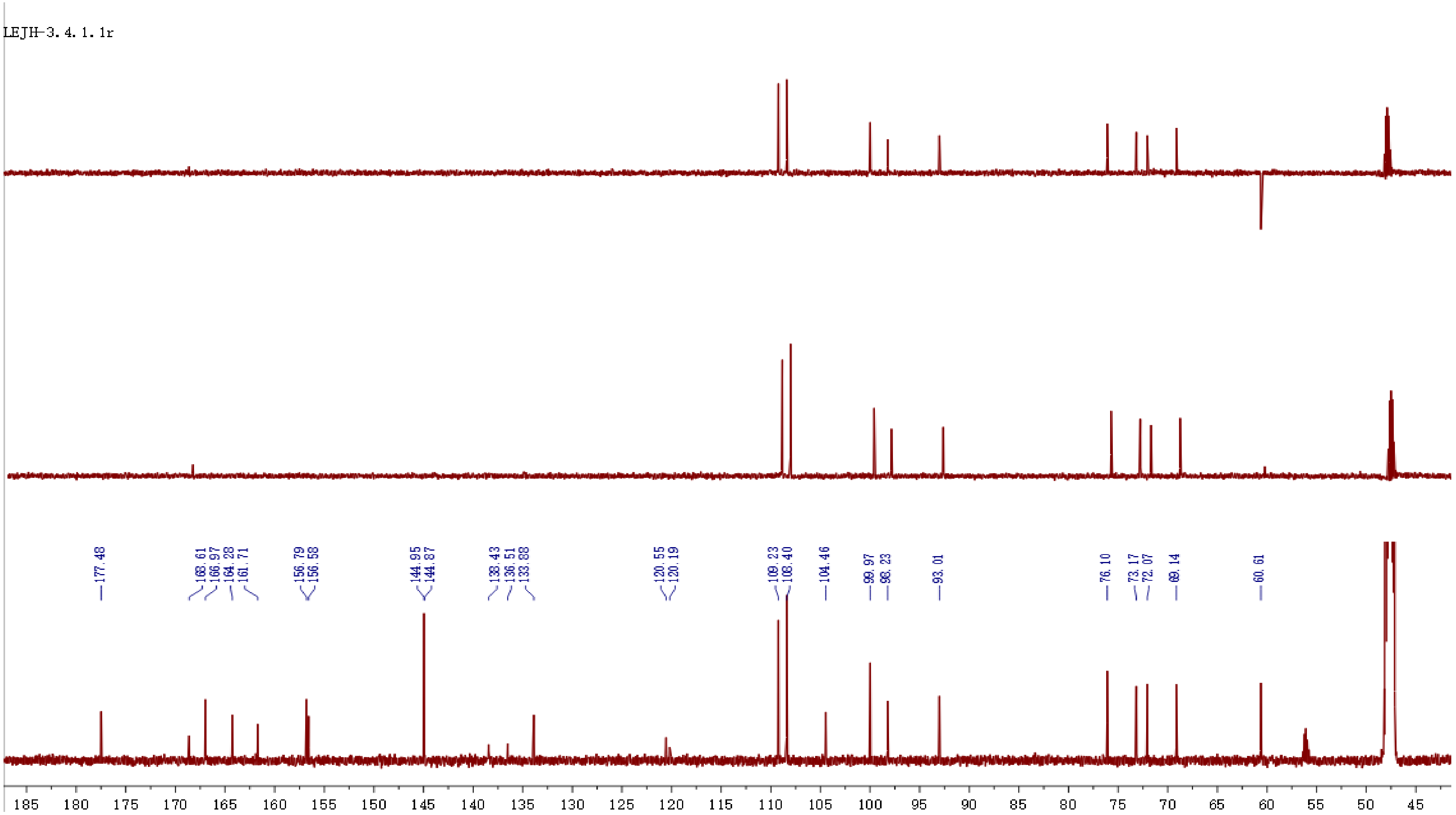

Figure S6 ^{13}C NMR spectrum of **3.**

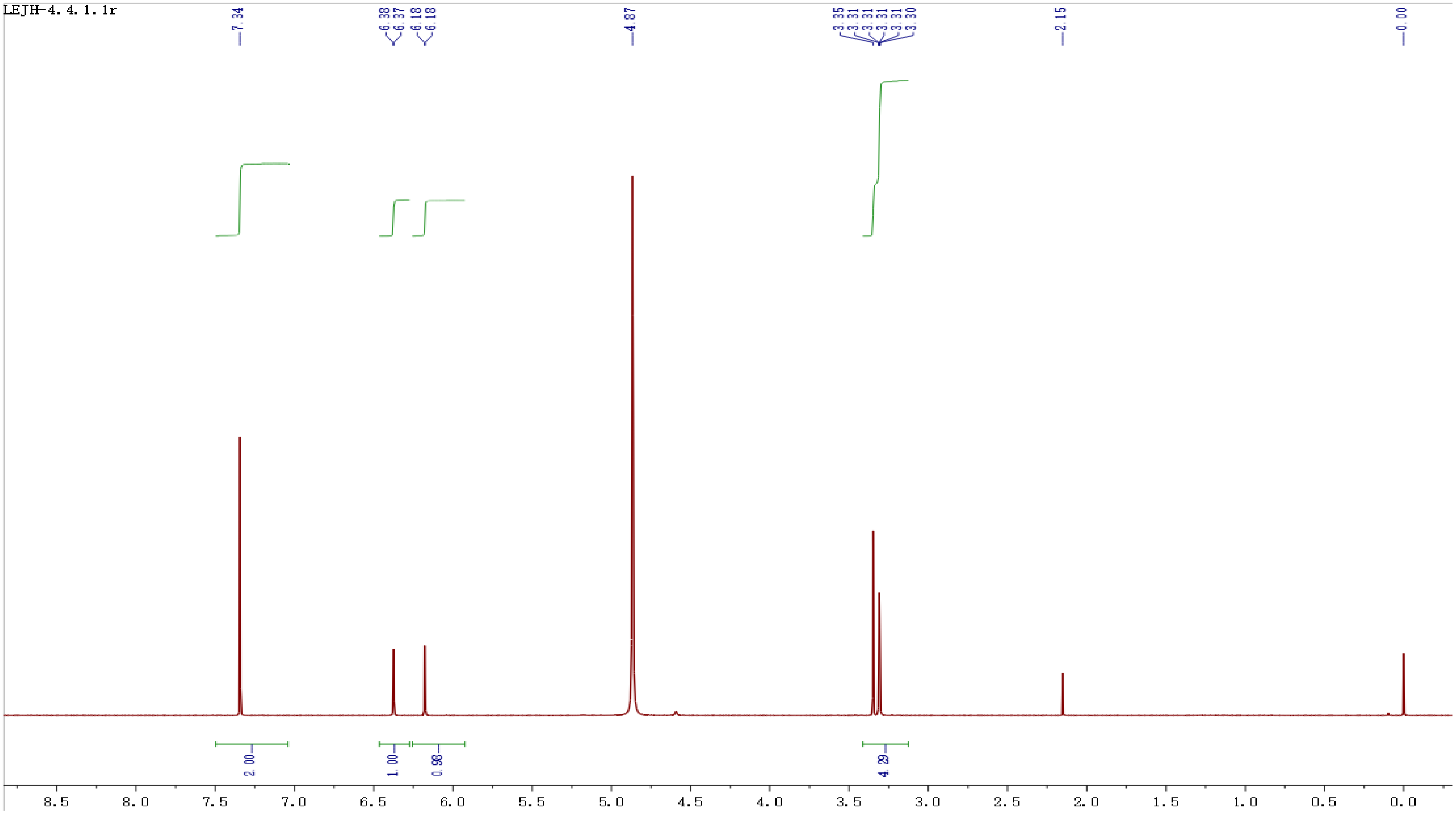

Figure S7 ^{1}H NMR spectrum of **4**.

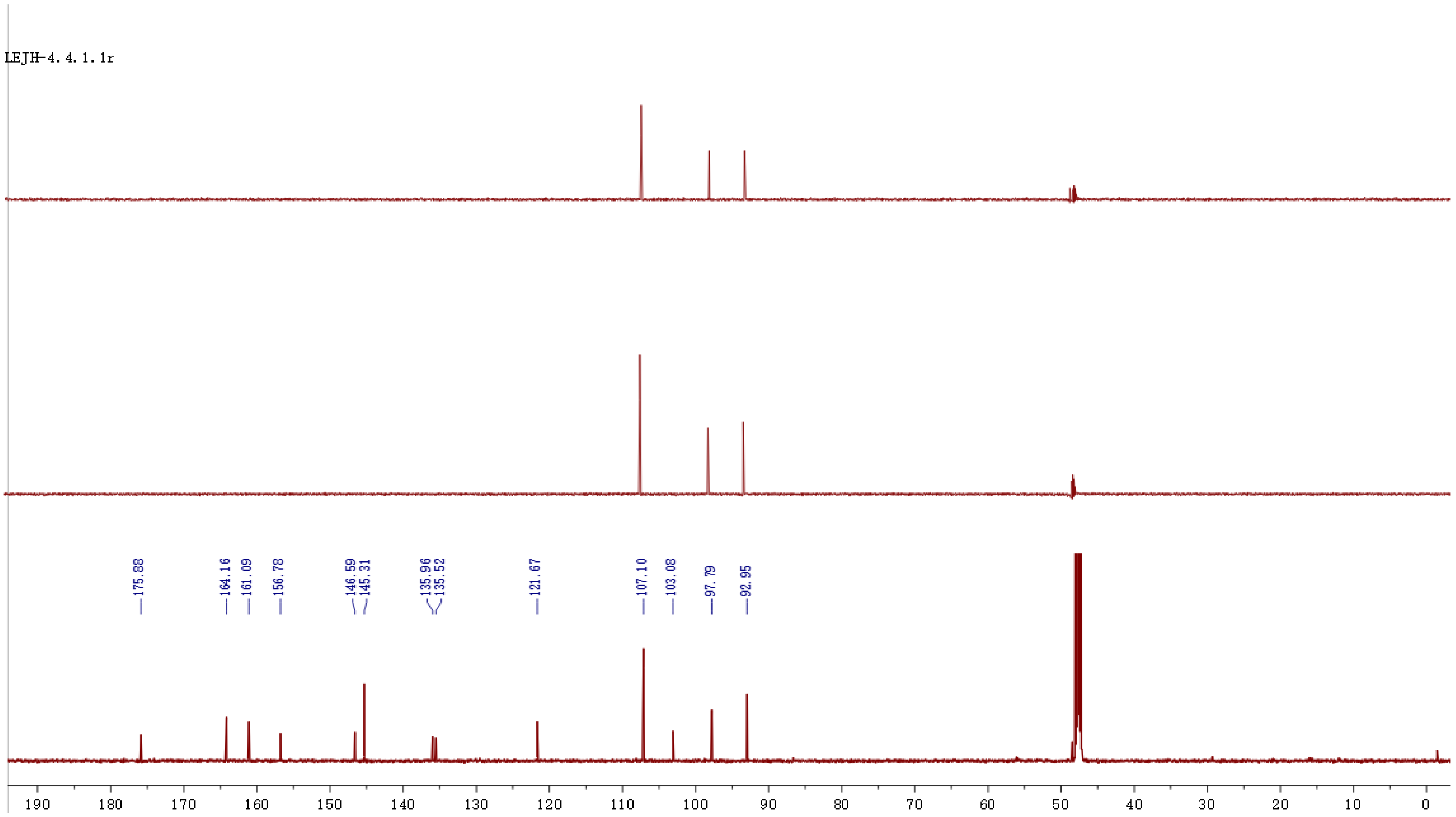

Figure S8 ^{13}C NMR spectrum of **4.**

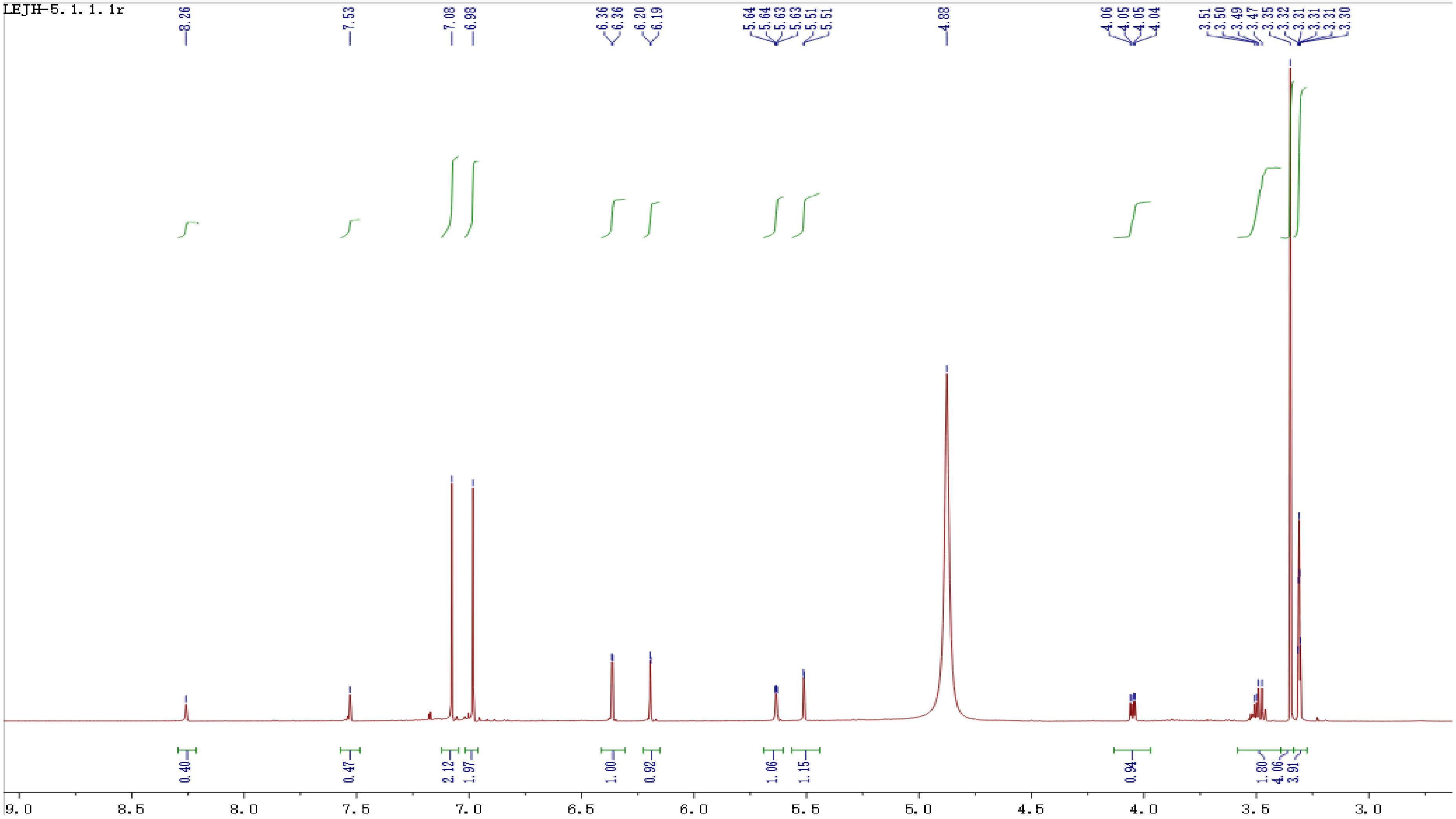

Figure S9 ^{1}H NMR spectrum of **5**.

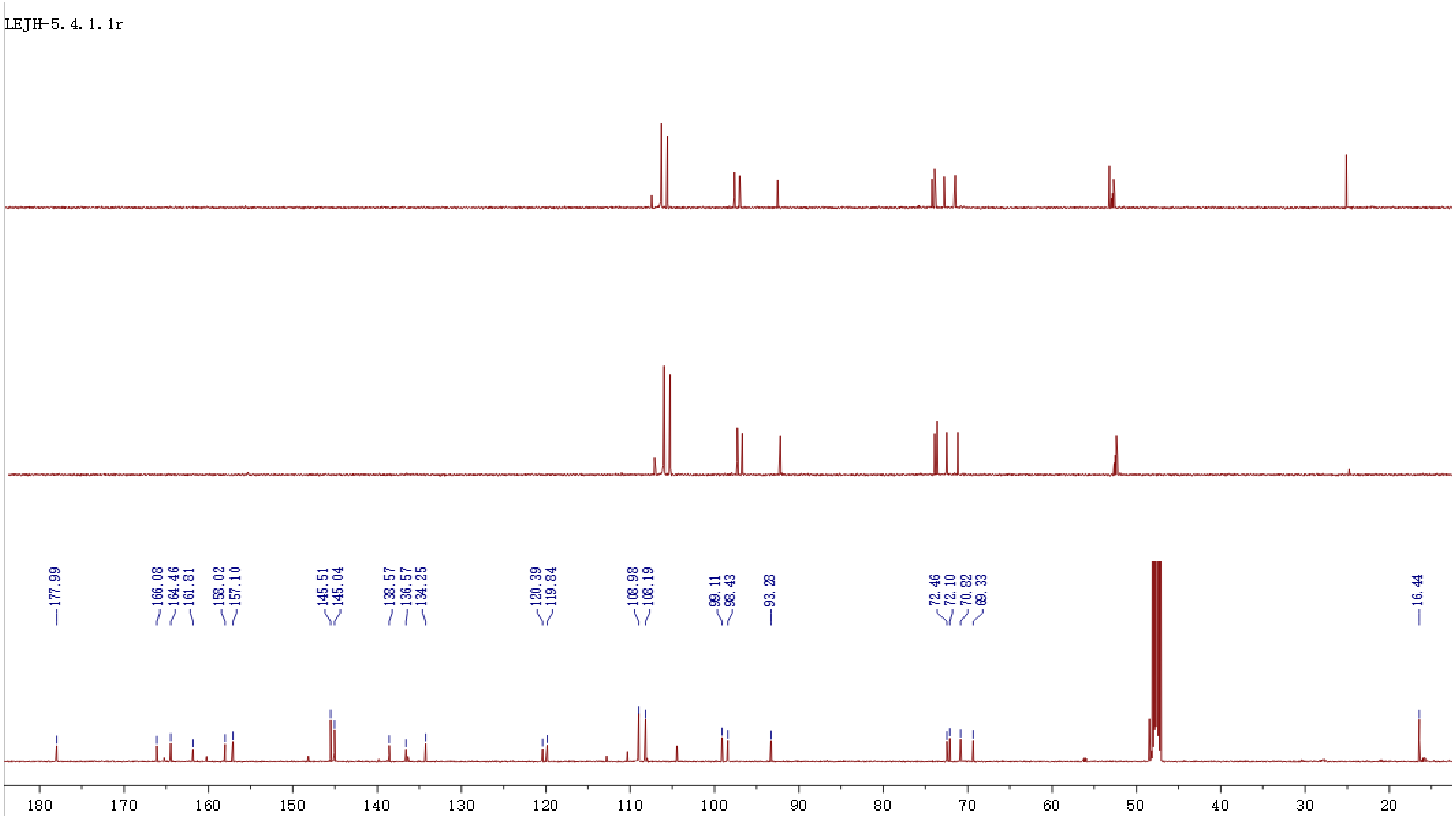

Figure S10 ^{13}C NMR spectrum of **5.**

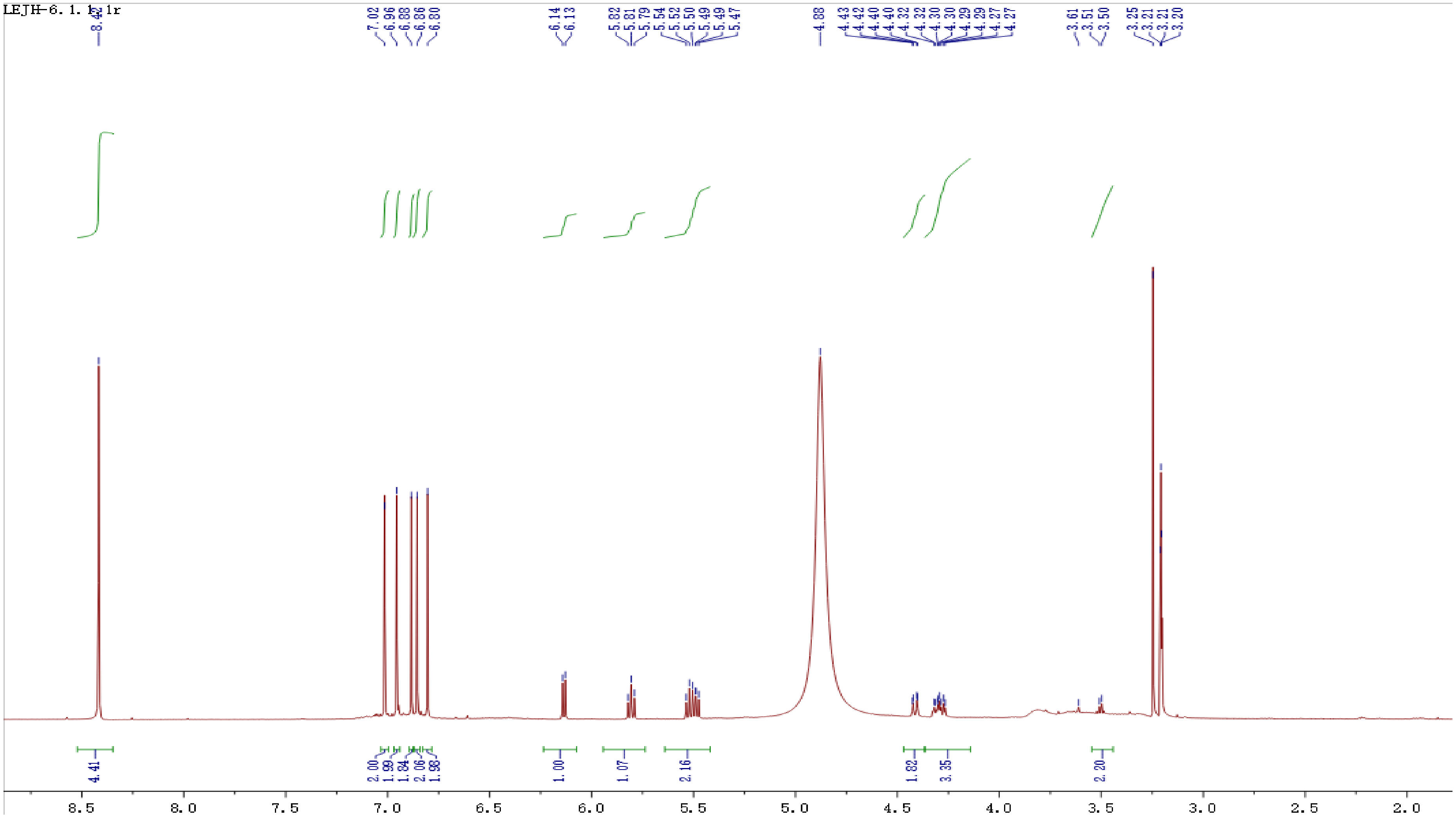

Figure S11 ^{1}H NMR spectrum of **6**.

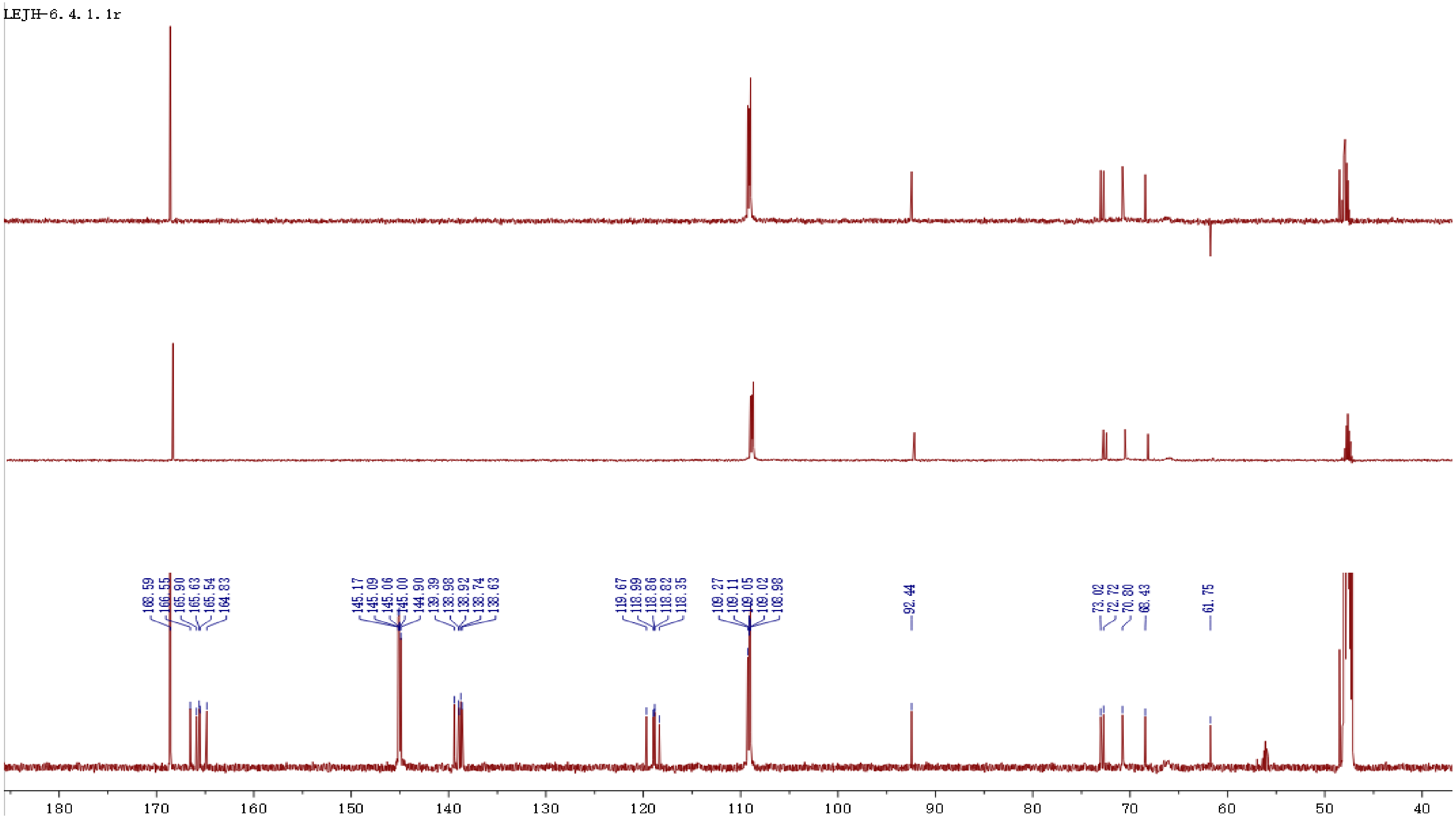

Figure S12 ^{13}C NMR spectrum of **6.**

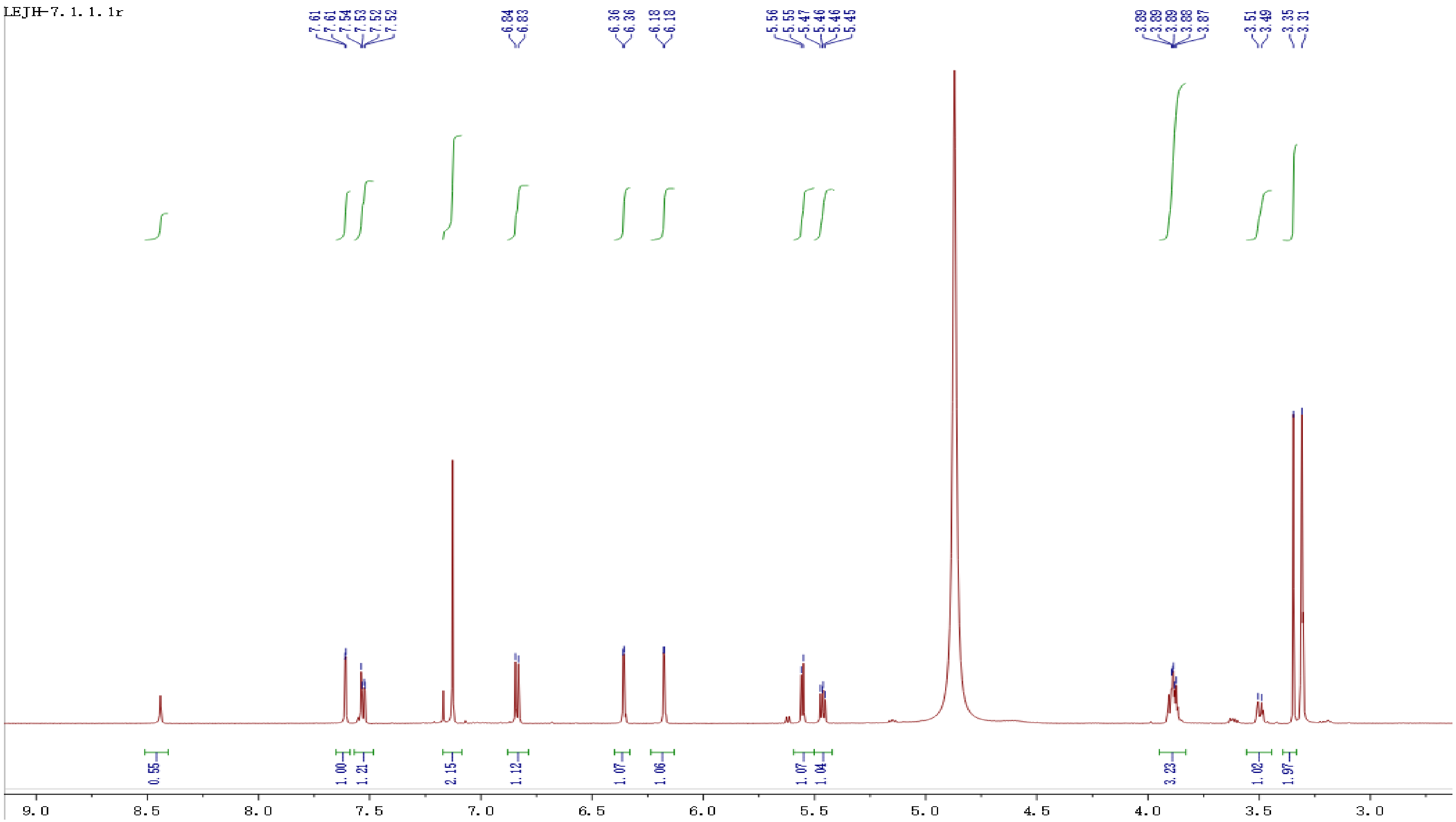

Figure S13 ^{1}H NMR spectrum of **7**.

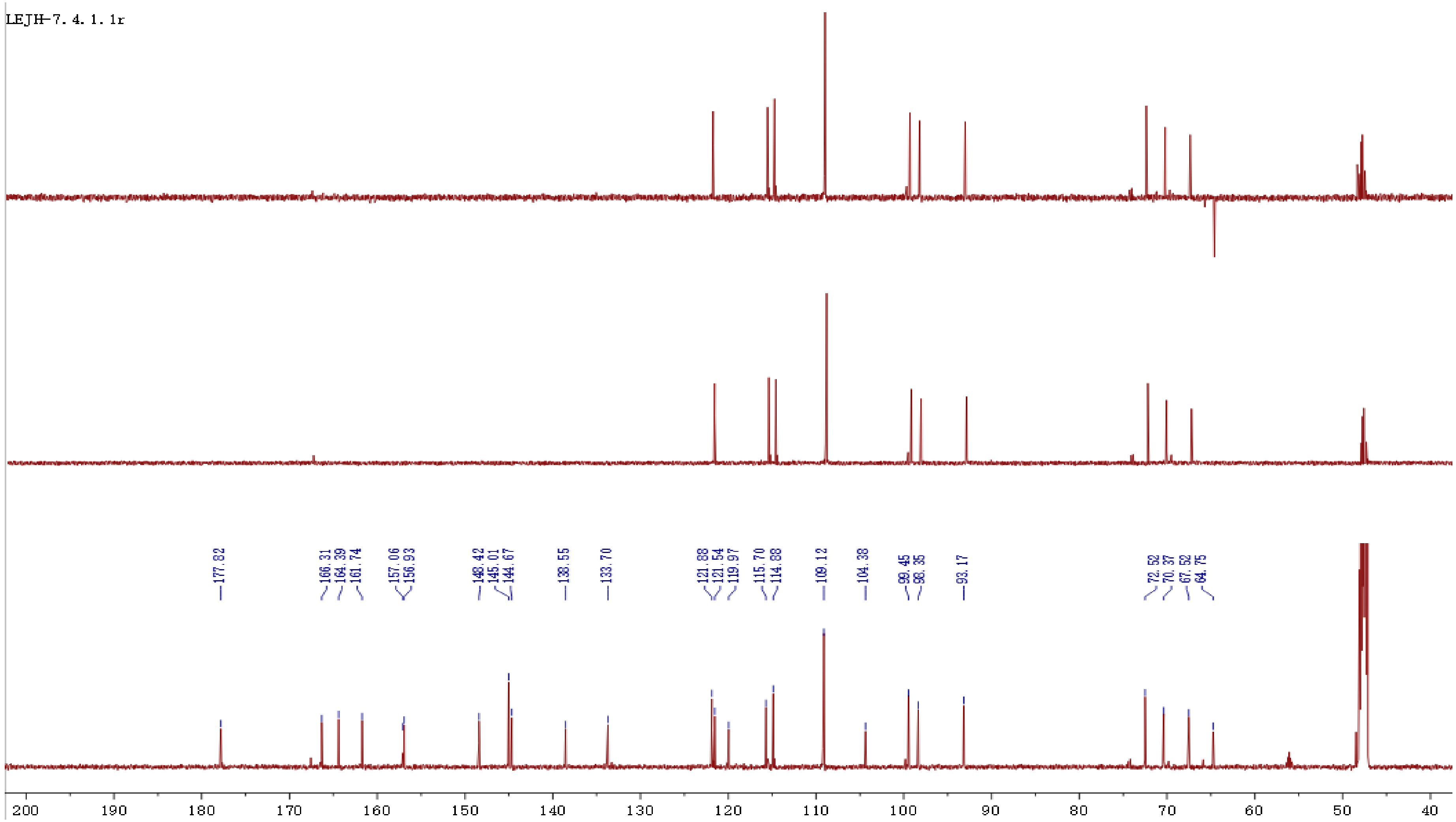

Figure S14 ^{13}C NMR spectrum of **7.**
